# Astrocytes and neurons share region-specific transcriptional signatures that confer regional identity to neuronal reprogramming

**DOI:** 10.1126/sciadv.abe8978

**Published:** 2021-04-07

**Authors:** Álvaro Herrero-Navarro, Lorenzo Puche-Aroca, Verónica Moreno-Juan, Alejandro Sempere-Ferràndez, Ana Espinosa, Rafael Susín, Laia Torres-Masjoan, Eduardo Leyva-Díaz, Marisa Karow, María Figueres-Oñate, Laura López-Mascaraque, José P. López-Atalaya, Benedikt Berninger, Guillermina López-Bendito

**Affiliations:** 1 Instituto de Neurociencias, Universidad Miguel Hernández-Consejo Superior de Investigaciones Científicas (UMH-CSIC), Sant Joan d’Alacant, Spain.; 2 Institute of Psychiatry, Psychology, and Neuroscience, Centre for Developmental Neurobiology, and MRC Centre for Neurodevelopmental Disorders, King’s College London, SE1 1UL London, UK.; 3 Institute of Biochemistry, Friedrich-Alexander-University Erlangen-Nürnberg, 91054 Erlangen, Germany.; 4 Physiological Genomics, Biomedical Center, Ludwig Maximilians University Munich, 82152 Planegg/Munich, Germany.; 5 Instituto Cajal, CSIC, Madrid, Spain.; 6 Institute of Physiological Chemistry, University Medical Center Johannes Gutenberg University Mainz, 55128 Mainz, Germany.

## Abstract

Neural cell diversity is essential to endow distinct brain regions with specific functions. During development, progenitors within these regions are characterized by specific gene expression programs, contributing to the generation of diversity in postmitotic neurons and astrocytes. While the region-specific molecular diversity of neurons and astrocytes is increasingly understood, whether these cells share region-specific programs remains unknown. Here, we show that in the neocortex and thalamus, neurons and astrocytes express shared region-specific transcriptional and epigenetic signatures. These signatures not only distinguish cells across these two brain regions but are also detected across substructures within regions, such as distinct thalamic nuclei, where clonal analysis reveals the existence of common nucleus-specific progenitors for neurons and astrocytes. Consistent with their shared molecular signature, regional specificity is maintained following astrocyte-to-neuron reprogramming. A detailed understanding of these regional-specific signatures may thus inform strategies for future cell-based brain repair.

## Teaser

Region-specific gene expression shared with neurons imparts to astrocytes competence for region-specific neuronal reprogramming.

## INTRODUCTION

The development of neuronal diversity is central for the organization and function of the central nervous system (CNS). This diversity is largely determined by specific transcriptional programs already expressed at the progenitor stage (*1*–*7*). These programs can undergo temporal regulation, allowing for sequential generation of different progeny from the same original progenitor (*4*, *8*). The most drastic case of this temporal regulation occurs at the switch of progenitors from neurogenic to gliogenic competence (*9*). Moreover, transcriptional programs are also diversified across brain regions to reflect the positional identity of the progenitors. Pioneering work in the spinal cord suggests that the diversification of astrocytes might follow the same organizing principle of positional identity (*10*, *11*). This notion has recently received further support by clonal analyses and single-cell transcriptomics that unveiled highly characteristic distributions of heterogeneous astroglia within and across brain regions (*12*–*15*). However, given that neurons and astroglia are generated from the same germinal zones, they could share common molecular signatures, reflecting their origin and potentially acting to coordinate region-specific developmental features. Here, we address this possibility and report that thalamic and cortical astrocytes exhibit region-specific transcriptional and epigenetic signatures, which are shared with the neurons generated within the same thalamic or cortical progenitor domain but not beyond. These shared signatures confer a remarkable degree of regional specification for astrocyte-to-neuron reprogramming induced by the proneural factor Neurogenin 2. Last, manipulating the regional-specific code in defined astrocyte populations redirects reprogramming toward neurons of different, yet predictable, regional identity.

## RESULTS

### Shared gene expression signatures between astroglia and neurons

**Fig. 1:**
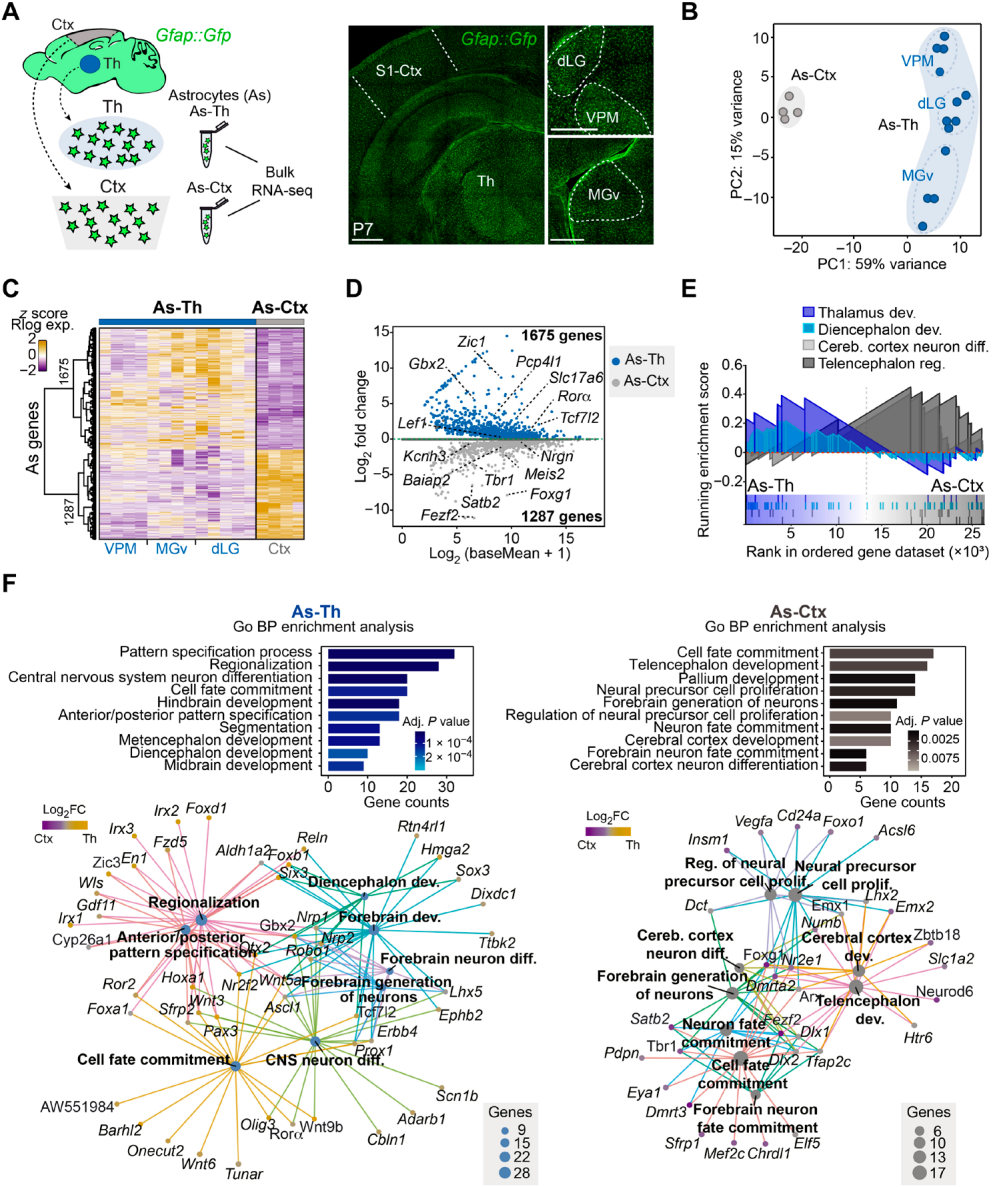
Astrocytes show region-specific transcriptomic profiles. (**A**) Schematic of the RNA-seq experiments for cortical and thalamic astrocytes. Astrocytes from P7 *Gfap::Gfp* mice were fluorescence-activated cell sorting (FACS)–purified and sequenced. Right, images showing the thalamus and cortex of a *Gfap::Gfp* mouse at P7. (**B**) Principal components analysis (PCA) of the transcriptomes of astrocytes (As) from the thalamus (Th), including dLG (*n* = 5 samples), VPM (*n* = 4), and MGv (*n* = 4), and cortex (Ctx, *n* = 4) at P7. (**C**) Heatmap of *z* score normalized regularized logarithm (Rlog) expression and unbiased clustering of significantly DEGs between thalamic (As-Th) and cortical astrocytes (As-Ctx). Each row represents a gene, the columns are biological replicates, and the color code represents the normalized expression for up-regulated genes in yellow versus down-regulated genes in purple. (**D**) MA plot displaying DEGs. Blue and light gray dots represent thalamic and cortical DEGs with their mean normalized counts, respectively. Dark gray dots represent genes that failed to give a significant result. (**E**) Enrichment plots from the GSEA of two specific GO terms related to the thalamic and cortical formation. (**F**) GO biological process (BP) enrichment analysis of significantly DEGs in thalamic and cortical astrocytes and associated gene networks. The size of every node (enriched term) represents the number of genes enriched and the color code (yellow, high expression; purple, low expression) corresponds to the log_2_FC in DE analysis. In (A), scale bars, 400 μm.

**Fig. 2:**
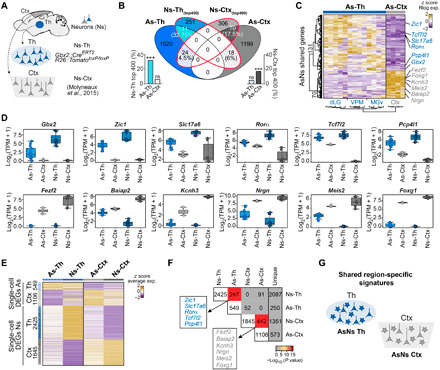
Astrocytes and neurons share region-specific transcriptome profiles. (**A**) Schematic of the RNA-seq experiments for comparing thalamic and cortical neurons. Thalamic neurons were obtained from *Gbx2-Cre::Tomato-floxed* P0 mice and cortical neurons from publicly available datasets (*20*). Ns-Th included dLG (*n* = 4), VPM (*n* = 4), and MGv (*n* = 3), and Ns-Ctx (*n* = 6). (**B**) Venn diagram showing the genes that overlap between astrocytes (As) and neurons (Ns) in both the thalamus and cortex. Bar plots represent the percentage of the enriched genes shared between populations. (**C**) Heatmap showing overlapping genes between As and Ns in the thalamus and cortex. (**D**) Box plots showing expression levels of selected region-specific genes shared between neurons and astrocytes of the thalamus (top) or the cortex (bottom). TPM, transcripts per million. (**E**) Heatmap of the *z* score of average expression levels of DEGs at the single-cell level, identified by comparing cell types among different regions of origin (As-Th versus As-Ctx; Ns-Th versus Ns-Ctx) from publicly available data (*23*). (**F**) Comparison matrix of the number of shared specific gene lists between As and Ns datasets of every specific region. Color code according to significance of overlap. (**G**) Schematic of the main conclusion of the experiments. Data are plotted with box-and-whisker plots, which give the median, 25th and 75th percentiles, and range. Dots in (D) represent every single value.

To test the hypothesis that astrocytes and neurons generated within the same brain region share molecular signatures unique to this region, we set out to identify differentially expressed genes (DEGs) between astrocytes of the thalamus and cortex, performed a similar analysis between thalamic and cortical neurons, and then searched for potential overlap among the two sets of DEGs. Toward this, we performed bulk RNA sequencing (RNA-seq) on astrocytes isolated from the thalamus [comprising dorsolateral geniculate (dLG), ventral posteromedial (VPM), and ventromedial geniculate (MGv) nuclei] and primary somatosensory cortex (S1) using astrocyte reporter mice (*Gfap::Gfp*) (*16*) at postnatal day 7 (P7) (Fig. 1A and fig. S1A) after the peak of astrogenesis (*17*). As for the analysis of neurons, we performed RNA-seq on neurons isolated from the thalamus at P0 using a *Gbx2-CreER::Tomato-floxed* thalamic reporter mouse, where early postmitotic thalamic neurons are labeled (fig. S1B) (*18*). By intersectional analysis within these astrocytic datasets, we first identified genes specifically expressed by astrocytes irrespective of their region of origin (e.g., *Aqp4* and *Aldh1l1*; fig. S1C). As for neurons, we used canonical genes conserved in all neuronal subtypes (e.g., *Rbfox3* or *Nefm*; fig. S1C) [see (*19*–*21*)]. The unambiguous expression pattern of these genes support the specificity of the *Gfap::Gfp* and *Gbx2-CreER::Tomato-floxed* mouse lines used for isolation of astrocytes and thalamic neurons, respectively (fig. S1, A to D). A principal components analysis (PCA) revealed that thalamic and cortical astrocytes clustered according to their anatomical origins (Fig. 1B). Moreover, a differential expression analysis (DEA) revealed 1675 versus 1287 DEGs enriched in the thalamus and cortex, respectively (Fig. 1C). Among the DEGs enriched in each population, we identified several genes, including transcription factors, that are known to be highly expressed in neurons of the respective regions (Fig. 1D and table S1) (*20*, *22*). This prompted us to perform Gene Ontology (GO) overrepresentation and gene set enrichment analyses (GSEA) of the DEGs between thalamic and cortical astrocytes, which revealed marked differences in developmental programs and distinct region-specific molecular pathways that have been previously associated with neurons from these regions (Fig. 1, E and F). To unveil region-specific genes shared among astrocytes and neurons of the corresponding regions, we first identified the most highly DEGs enriched in thalamic and cortical neurons, by comparing RNA-seq data of neurons isolated at P0 from a thalamic reporter line (*Gbx2-CreER::Tomato-floxed*) (*18*) with a published dataset of P1 cortical neurons (Fig. 2A) (*20*). We found that genes specifically enriched in thalamic or cortical neurons were substantially overrepresented among DEGs in thalamic or cortical astrocytes, respectively. Among the 400 most DEGs in thalamic neurons, only 6% were shared with cortical astrocytes, whereas 32.75% of these genes were significantly expressed by thalamic astrocytes, albeit typically at a lower level (including *Gbx2*, *Ror*α, or *Tcf7l2*; Fig. 2, B to D, and fig. S1, E to K). A significant overlap in gene expression was also observed for cortical neurons and cortical astrocytes (17.5%), where genes such as *Fezf2* or *Foxg1* were identified in both populations. Overlap in gene expression was notably lower between cortical neurons and thalamic astrocytes (4.5%; Fig. 2, B to D).

Next, we interrogated the overlap in expression of region-specific genes between neurons and astrocytes at the single-cell level by analyzing an independent, published dataset containing single-cell transcriptomes of thalamic and cortical neurons and astrocytes from juvenile/young adult mice (fig. S2; fig. S3, A and B; and table S2) (*23*). The analysis of these single-cell data fully confirmed the existence of region-specific gene expression programs shared between astrocytes and neurons of thalamic and cortical origin, respectively (Fig. 2, E to G, and fig. S3, C to G). Notably, among the DEGs at the single-cell level shared between neurons and astrocytes (247 for the thalamus and 442 for the cortex), we found numerous genes known to confer regional neuronal identity (e.g., *Ror*α, *Tcf7l2*, *Fezf2*, or *Foxg1*), as observed in the bulk RNA-seq dataset. These single-cell data demonstrate that the shared region-specific transcriptional signature is not a transient developmental feature but maintained well beyond the first postnatal week.

Last, we conducted two additional experiments to validate the expression of region-specific “neuronal” genes in astrocytes. First, we performed fluorescence in situ hybridization (FISH) in fixed slices from *Gfap::Gfp* brains at P7 and confirmed the expression, in a region-specific manner, of shared genes in astrocytes (GFP^+^ cells) (fig. S4, A and B). This expression was mainly found at the level of mRNA, as the corresponding proteins were only detected in a low percentage of the astrocytes, at least for the genes analyzed (fig. S4C), which suggests that posttranscriptional regulations might take place (*24*). Second, we isolated, purified, and cultured astrocytes from the thalamus or cortex and performed quantitative polymerase chain reaction (qPCR) for region-specific genes, confirming the expression of shared genes in astrocytes (fig. S4, D and E). Thus, single-cell RNA-seq (scRNA-seq), FISH, and qPCR provide strong support for the specificity of the detection of neuronal genes in astrocytes, arguing against neuronal contamination of the astrocyte datasets.

**Fig. 3:**
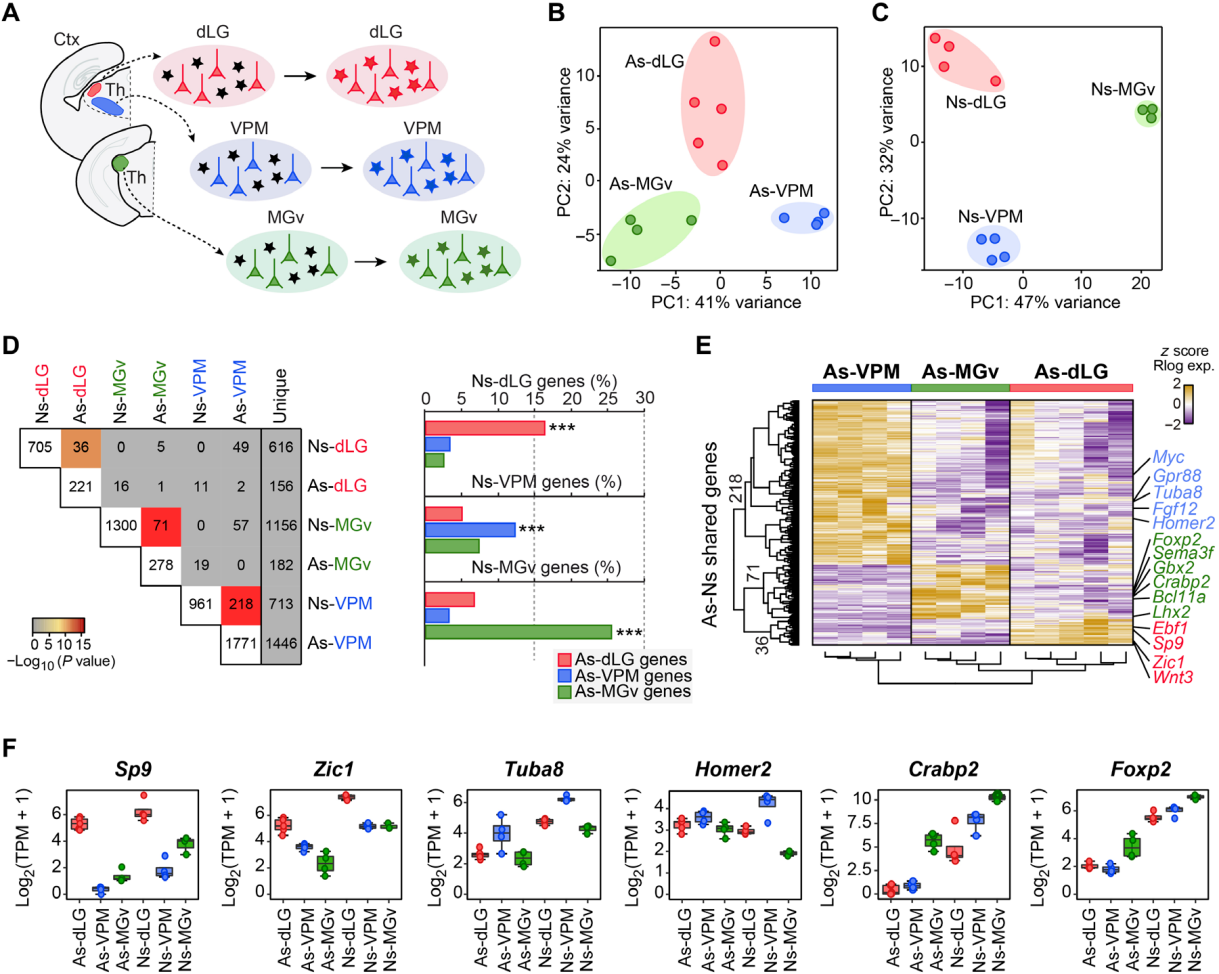
Sensory-modality thalamic astrocytes and neurons express common specific genes for every nucleus. (**A**) Schematic of the RNA-seq experiments for comparing neurons and astrocytes from the sensory thalamic nuclei (dLG, VPM, and MGv) and main conclusion obtained. (**B**) PCA of transcriptomes from astrocytes (As) isolated from the distinct sensory-modality thalamic nuclei [dLG (*n* = 5), VPM (*n* = 4), and MGv (*n* = 4)] at P7. (**C**) PCA of transcriptomes of neurons (Ns) from the distinct sensory-modality thalamic nuclei [dLG (*n* = 4), VPM (*n* = 4), and MGv (*n* = 3)] at P0. (**D**) Left, comparison matrix of the number of shared specific gene lists between neurons and astrocytes datasets of every thalamic nuclei. Color code according to significance of overlap. Right, bar plots representing the percentage of gene overlap between As and Ns from each thalamic nucleus. (**E**) Heatmap showing the overlapping DEGs between As and Ns in each nucleus. Each column represents a biological replicate and the color code represents the *z* score normalized expression (up-regulated genes in yellow, down-regulated genes in purple). (**F**) Box plots showing expression levels of nuclei-specific shared genes between astrocytes and neurons in the distinct sensory-modality thalamic nuclei. TMP, transcripts per million. Data are plotted with box-and-whisker plots, which give the median, 25th and 75th percentiles, and range. Dots in (F) represent every single value. ***P < 0.0005.

We next asked whether region-specific gene expression programs can be identified at the level of individual regional subdivisions such as those of sensory thalamic nuclei. Thus, we compared the transcriptomes of astrocytes and neurons from the three main sensory thalamic nuclei (dLG, VPM, and MGv; Fig. 3A). PCA identified three well-defined clusters corresponding to each nucleus in both astrocytes and neurons, supporting the notion that the identity of each thalamic nucleus is encoded transcriptionally in a cell type–independent manner (Fig. 3, B and C). Hence, the nucleus-specific DEGs of astrocytes exhibited a significant overlap with those of the neurons from the same nucleus (e.g., *Sp9* for dLG or *Crabp2* for MGv; Fig. 3, D and E, and tables S3 and S4), although the expression levels of these genes were notably lower in the astrocytic populations (Fig. 3F). Our single-cell data analysis also revealed a region-specific pattern of shared genes between astrocytes and neurons along the anteroposterior axis of the cerebral cortex (fig. S5 and table S5), supporting a generalization of the existence of region- and subregion-specific shared transcriptional programs between these two major cell types in the mouse brain.

### Thalamic progenitor clones are nucleus specific

**Fig. 4:**
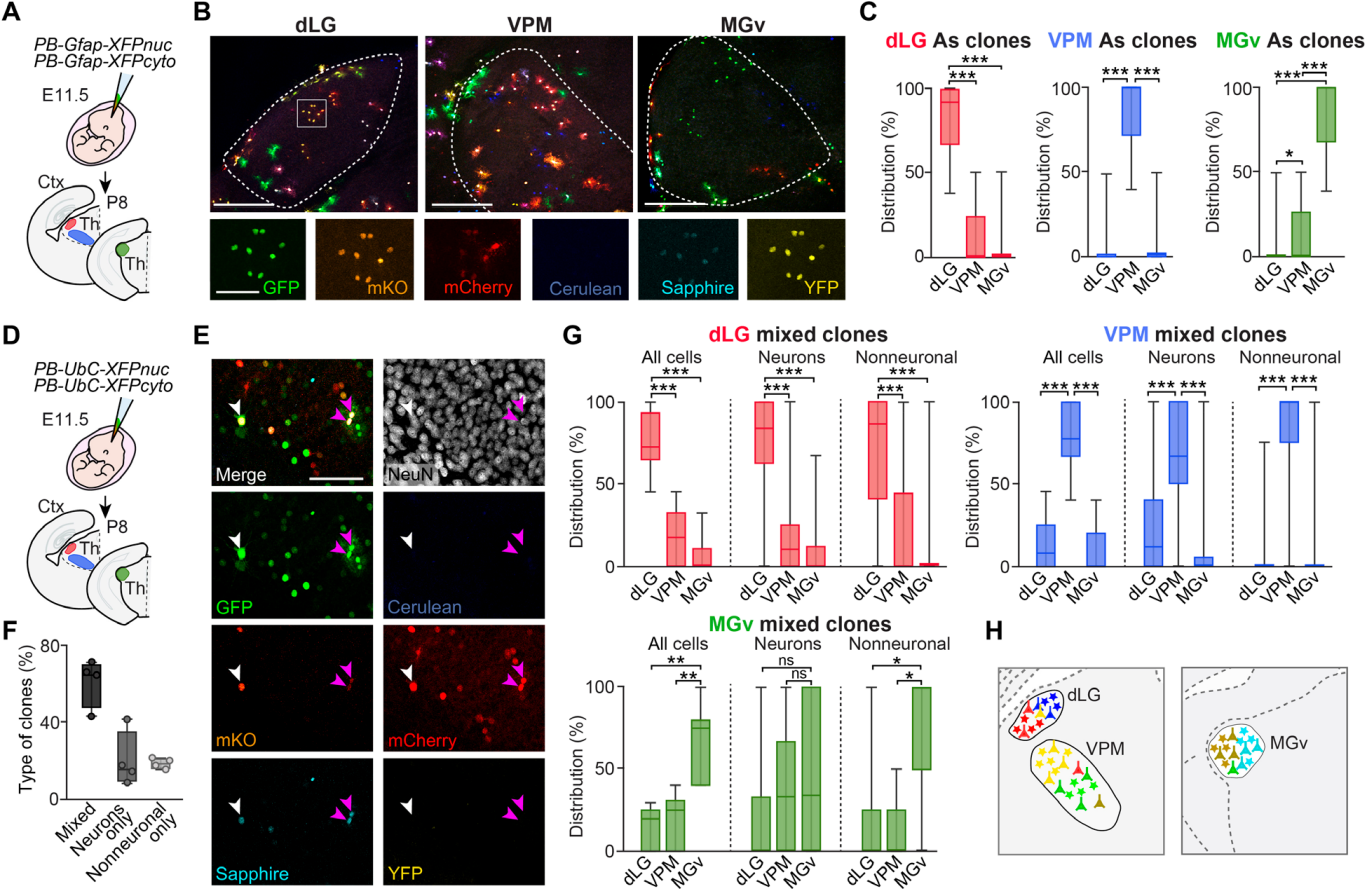
Clonally related astrocytes and neurons remain within the same nuclear boundaries. (**A**) Experimental design for the analysis of astrocytic clones in the sensory nuclei (dLG, VPM, and MGv). A cocktail of integrative plasmids encoding six different fluorescent proteins under the Gfap promoter (GFAP-*StarTrack*) was electroporated in the third ventricle at E11.5. (**B**) Thalamic astrocytes labeled with the GFAP*-StarTrack* constructs at P8. Insets show the expression of each fluorescent reporter in a dLG astrocyte clone (white square). (**C**) Quantification of the dispersion of the clonally related astrocytes (*n* = 320 clones from five electroporated animals). (**D**) Experimental design for the study of neuronal and nonneuronal clonal cells with the UbC*-StarTrack* constructs. (**E**) Example of a neuron (white arrows) and two astrocytes (purple arrows) from the VPM coming from the same progenitor, thus sharing the same color code. (**F**) Three types of clones were analyzed clones based on their cell-type composition: mixed clones (containing neurons and nonneuronal cells), clones with neurons only, or clones with nonneuronal cells only (*n* = 4 electroporated animals). (**G**) Quantification of the dispersion of clonally related neuronal and nonneuronal cells from mixed clones, in the different thalamic sensory nuclei (*n* = 130 clones from four electroporated animals). (**H**) Schema representing the specificity in the nuclei-dependent localization of clonal cells. Cells coming from the same progenitor are colored with the same color. Note that most clonally related cells respect the nuclei segregation and only few cells are dispersed. Data are plotted with box-and-whisker plots, which give the median, 25th and 75th percentiles, and range. Scale bars, 100 μm. ns, not significant; *P < 0.05, **P < 0.005, and ***P < 0.0005.

Next, we investigated whether the significant gene expression overlap between postmitotic astrocytes and neurons from the same region reflects a common clonal origin during embryonic development. This would imply that within the thalamus, cells belonging to the same clone should not disperse beyond nuclear boundaries. To test this hypothesis, we first analyzed the distribution of astrocytes originating from single clones across thalamic sensory nuclei. We tracked astrocyte clones arising from embryonic day 11.5 (E11.5) progenitors by electroporating a battery of plasmids encoding distinct fluorophores under the control of the *Gfap* promoter, following transposase-mediated integration (“StarTrack”) (*12*), and analyzed the dispersion of each clone at P8 (Fig. 4, A and B, and fig. S6A). This revealed that clonally related astrocytes remain within the boundaries of a given nucleus with little dispersion to other nuclei, even in the case of larger clones (>10 cells) (Fig. 4C and fig. S6, B and C). Next, we addressed the question of whether thalamic progenitors that generate astrocytes also produce neurons and, if so, whether these neurons stay within the same nuclear boundaries as their sibling astrocytes. Thalamic clones containing neuronal and/or nonneuronal cells were tracked by using the same set of fluorophores under the control of a ubiquitously expressed promoter (Fig. 4, D and E, and fig. S6, D and E) (*25*). While we found 39% of clones consisting only of neurons or glia, the majority (61%) were mixed, containing similar proportions of neurons and glia (Fig. 4F and fig. S6F). We found that mixed clones covered territories that largely respected nuclear boundaries, although neurons exhibited a wider range of dispersion (Fig. 4, G and H, and fig. S6G), extending and confirming previous studies (*26*, *27*). Our data suggest that the overlap in region-specific gene expression between neurons and astrocytes of each sensory thalamic nucleus is the result of their common clonal origin together with the limited spatial dispersion of clonally related cells and may indicate that positional information is retained from an early progenitor stage onward.

### Astrocytes reprogram into region-specific neurons

**Fig. 5:**
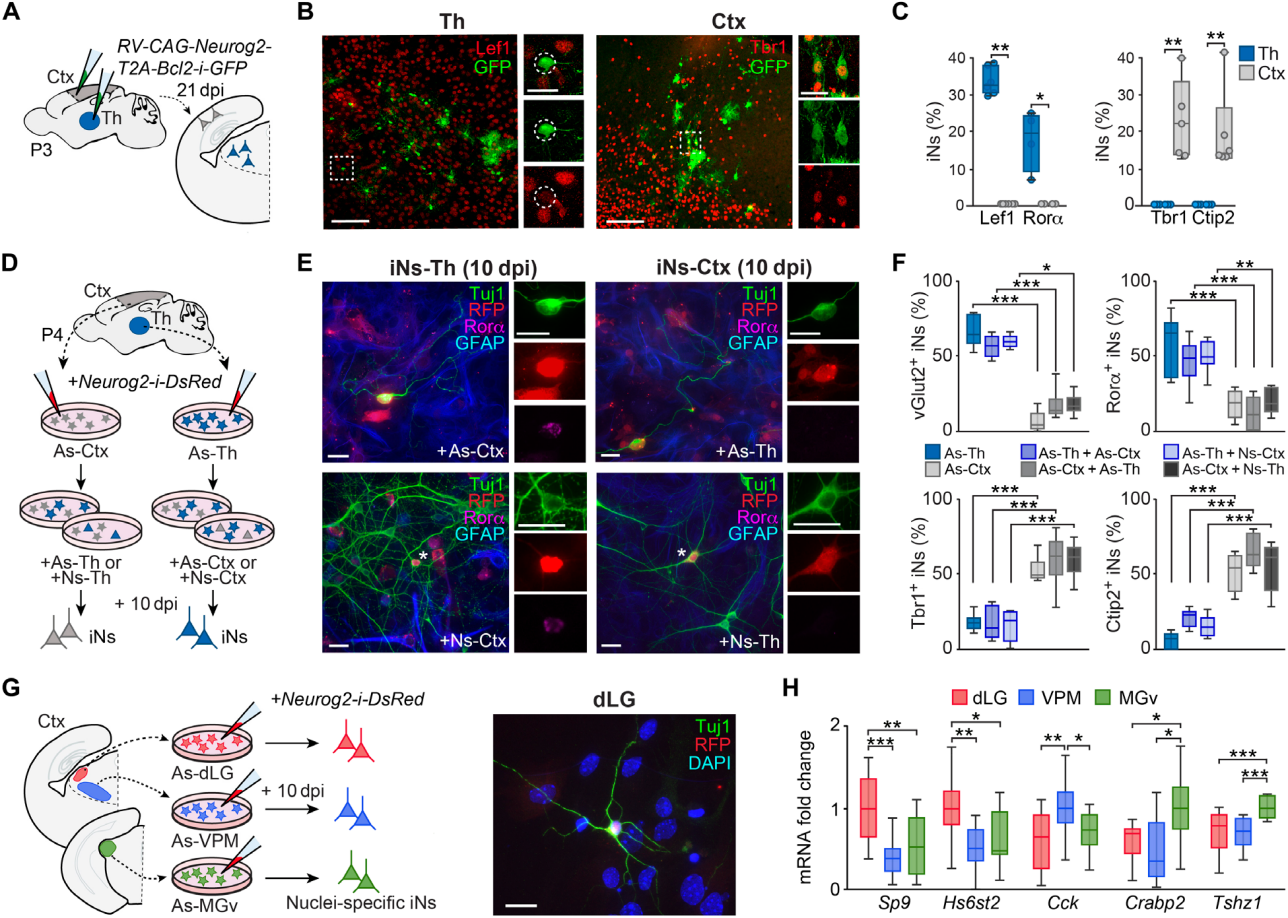
Astrocytes are reprogrammed into region-specific neurons. (**A**) Experimental design for the in vivo reprogramming. Retrovirus encoding *Neurog2* and *Bcl2* or only *Bcl2* were injected in the thalamus and cortex of P3 animals. (**B**) Immunofluorescence for thalamic and cortical markers in iNs reprogrammed from cortical or thalamic astrocytes in vivo. (**C**) Percentage of iNs expressing thalamic or cortical markers after reprogramming in vivo (*n* = 4 to 6 injected mice). (**D**) Experimental design for assessing the influence of the environment on the induced neurons identity. Isolated cortical or thalamic astrocytes were infected and then cocultured with thalamic or cortical astrocytes or neurons. (**E**) Immunostaining for the thalamic marker Rorα in cortical or thalamic iNs (RFP^+^/Tuj1^+^) in the different conditions. (**F**) Quantification of the percentage of iNs generated from cortical or thalamic astrocytes that express *vGlut2*, *Rorα*, *Tbr1*, or *Ctip2* in control conditions or when mixed with astrocytes or neurons from the thalamus or the cortex, respectively (*n* = 6 to 14 independent cultures per condition). (**G**) Left, experimental design. Astrocytes from dLG, VPM, and MGv were isolated, cultured, and infected with Neurog2 retrovirus. Right, image of an iN from dLG astrocytes at 10 days post infection (dpi). (**H**) Reverse transcription (RT)–qPCR showing the expression of specific neuronal genes in the iNs after 10 dpi (*n* = 10 to 14 independent cultures per condition). Data are plotted with box-and-whisker plots, which give the median, 25th and 75th percentiles, and range. Dots in (C) represent every single value. Scale bars, 100 μm in (B) (insets, 25 μm) and 25 μm in (E) and (G). *P < 0.05, **P < 0.005, and ***P < 0.0005.

Since forebrain astrocytes and neurons share region-specific gene expression, we hypothesized that such molecular signature could instruct transcription factor–induced reprogramming of astrocytes toward an identity akin to their sibling neurons. To test this hypothesis, we injected a retrovirus encoding the proneural gene *Neurogenin 2* (*Neurog2*) and the cell death regulator *Bcl2* (*28*) into the somatosensory cortex and thalamus of P3 mice (Fig. 5A). At this developmental stage, retroviruses only transduce proliferative glia (*17*). Transduction with a retrovirus encoding *Bcl2* and *Gfp* alone, as control, resulted in labeling of glial cells. In contrast, transduction with *Neurog2*- and *Bcl2*-encoding retrovirus resulted in the appearance of numerous induced neurons (fig. S7, A and B). Time course analysis of the transduced cells demonstrated the gradual reprogramming of glia into neurons via a doublecortin (DCX)–positive immature neuronal stage in vivo (fig. S7, C to H). At 3 days post infection (dpi), the vast majority of the transduced cells (GFP^+^) were positive for the astrocytic marker Aldh1l1 and negative for the neuronal markers NeuN and DCX. Furthermore, we found that more than 90% of the transduced cells were also positive for 5-bromo-2′-deoxyuridine (BrdU), revealing that they are proliferating cells at the time of retroviral transduction (fig. S7D). After 7 and 14 dpi, the number of transduced cells positive for DCX or NeuN increased progressively. These DCX- or NeuN-positive cells were also BrdU positive, demonstrating again that they had been generated by the time of retroviral transduction and that DCX expression was not a result of reexpression in embryonically generated neurons (fig. S7, E to H). Consistent with our hypothesis, in vivo induced neurons expressed markers specific for a thalamic (*Lef1* and *Ror*α) or cortical (*Tbr1* and *Ctip2*) neuronal identity despite the fact that they were induced with the same transcription factor (Fig. 5, B and C).

Our data suggest that reprogramming of astrocytes into region-specific neurons is a consequence of their shared gene expression through a common lineage. However, it does not exclude the possibility that region-specific reprogramming is influenced by environmental signals provided by other local cells. To test this, we cultured astrocytes from the thalamus and cortex and examined their newly acquired neuronal identity for region-specific gene expression following reprogramming by *Neurog2* (fig. S8, A and B). As observed in vivo, thalamic and cortical induced neurons exhibited signatures of the thalamus and cortex, respectively, as shown by the differential expression of thalamic markers such as *Slc17a6* (*vGlut2*), *Ror*α, *Gbx2*, *Pou2f2*, or *Lef1* or cortical markers such as *Tbr1* or *Ctip2* (fig. S8, C to G). To exclude a prominent role of the environment in specifying the regional identity of induced neurons, we cocultured thalamic or cortical astrocytes undergoing reprogramming with neurons or astrocytes from the cortex or thalamus, respectively. Neurons induced from thalamic astrocytes expressed thalamic markers, irrespective of whether they had been cultured alone or with cortical cells. Conversely, cortical astrocytes gave rise to neurons expressing cortical markers irrespective of the culture conditions (Fig. 5, D to F). These experiments revealed that the regional identity of induced neurons is largely cell autonomous.

Last, as astrocytes and neurons from distinct thalamic sensory nuclei share expression of nucleus-specific genes, we hypothesized that reprogramming of thalamic astrocytes may yield neurons with nucleus-specific signatures. To this end, we isolated and reprogrammed astrocytes from dLG, VPM, and MGv in vitro with Neurog2 (Fig. 5G). We found that induced neurons derived from dLG astrocytes expressed dLG-specific genes *Sp9* and *Hs6st2*, while those derived from MGv astrocytes expressed MGv-specific genes *Crabp2* and *Tshz1.* Last, induced neurons of VPM astrocyte origin expressed the VPM marker *Cck* (Fig. 5H) (*22*). Together, these results show that Neurog2 triggers specific neuronal gene expression in astrocytes that reflects their place of origin.

### *Gbx2* respecifies cortical astrocytes toward thalamic fate

**Fig. 6:**
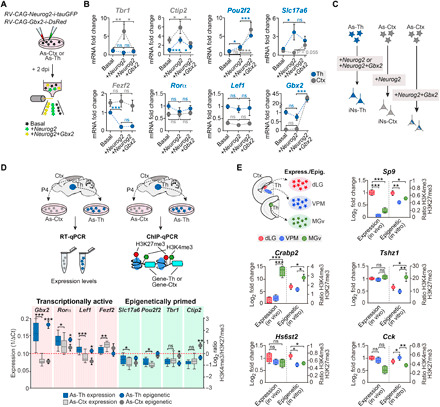
Poised epigenetic state of region-specific gene expression in astrocytes. (**A**) Experimental design. Astrocytes from the thalamus and cortex were cultured and infected with either *Neurog2-i-Gfp* retrovirus alone or both *Neurog2-i-Gfp* and *Gbx2-i-DsRed*. After 2 days, astrocytes were FACS-purified based on the presence of the reporter protein in three groups (noninfected, infected only with *Neurog2*, or infected with *Neurog2* and *Gbx2*). (**B**) Quantification of specific gene expression by RT-qPCR in astrocytes in basal conditions and 2 days after the overexpression of *Neurog2* alone or with a thalamic-specific gene (*Gbx2*) (*n* = 6 to 14 independent cultures per condition). Data are means ± SEM. (**C**) Schematic conclusion of the experiment. (**D**) Astrocytes from the thalamus and cortex were isolated, and the expression levels of some region-specific genes were assessed by RT-qPCR or ChIP-qPCR. Box-and-whisker plots represent the basal expression levels of the studied genes in thalamic and cortical astrocytes (left axis), and dots show the means ± SEM of the epigenetic state of the promoter of those genes, in terms of the presence of two histone marks, H3K4me3 and H3K27me3 (right axis) (*n* = 12 to 23 independent ChIP samples per condition). The red dashed line indicates the point where H3K4me3 and H3K27me3 marks are present at the same level. (**E**) Box-and-whisker plots showing the H3K4me3 and H3K27me3 ratio in vitro (left axis) (*n* = 14 to 18 independent ChIP samples per condition) and the basal in vivo expression of neuronal specific genes in thalamic astrocytes from each nucleus (right axis). ns, not significant; *P < 0.05, **P < 0.005, and ***P < 0.0005.

The aforementioned results strongly suggest that transcriptional signatures shared between neurons and astrocytes drive the regional specification of the latter during neuronal reprogramming. To directly test this, we examined whether coexpression of a thalamic fate determinant *Gbx2* (*29*), a factor being shared between astrocytes and neurons of the thalamus, could induce an early and fast redirection of neuronal reprogramming of cortical astrocytes toward a thalamic identity (Fig. 6A). Whereas in cortical astrocytes, expression of *Neurog2* for 2 days induced the expression of the cortical neuron fate determinants *Tbr1* and *Ctip2*, coexpression of *Gbx2* strongly suppressed this. Moreover, combined expression of *Neurog2* and *Gbx2* increased thalamic signature genes such as *Pou2f2* and *Slc17a6* (v*Glut2*) in cortical astrocytes (Fig. 6B). These data provide strong support for the partial redirection of neuronal reprogramming toward a thalamic identity (Fig. 6C). In thalamic astrocytes, by contrast, *Neurog2* sufficed for inducing significant expression of *Pou2f2* and *Slc17a6* expression (Fig. 6B). Genes that displayed differential regulation by Neurog2 with or without Gbx2 (*Slc17a6*, *Pou2f2*, *Tbr1*, and *Ctip2*) exhibited an epigenetically poised state in cortical or thalamic astrocytes, as determined by the ratio of active (H3K4me3) and repressive (H3K27me3) histone marks in their proximal regulatory elements (Fig. 6D and figs. S9, A to C, and S10, A and B). In contrast, nonresponsive genes (*Fezf2*, *Ror*α, and *Lef1*) exhibited origin-dependent baseline expression both transcriptionally and epigenetically in thalamic and cortical astrocytes (Fig. 6, B to D; fig. S9, A to C; and fig. S10, A and B).

Last, we addressed the question of whether a similar epigenetically poised state might explain the differential induction of nuclei-specific neuronal genes in astrocytes of distinct thalamic territories. To this end, we first compared basal expression levels and presence of active (H3K4me3) and repressive (H3K27me3) epigenetic marks at proximal regulatory elements of these genes, known to be differentially expressed in dLG, VPM, and MGv neurons (Fig. 3) (*22*). Intriguingly, irrespective of the baseline expression level, these genes exhibited an active (*Sp9*, *Crabp2*, and *Tshz1*) or poised/less repressed (*Hs6st2* and *Cck*) epigenetic state of their proximal regulatory elements, consistent with their nuclear origin (Fig. 6E and figs. S9D and S10, C and D). Nucleus-specific epigenetic priming might explain the observed differential transcriptional responsiveness to Neurog2 of genes whose levels of transcription are indistinguishable across nuclei before reprogramming. Future genome-wide analysis will be required to reveal the general importance of epigenetically poised states in dictating the region-specific gene expression following reprogramming.

## DISCUSSION

Using genome-wide analysis, we show that astrocytes of different brain regions actively transcribe genes that also correspond to regional genes in neurons. This remarkable relatedness between astrocytes and neurons from the same brain region correlates with their shared clonal origin, as shown for distinct sensory nuclei of the thalamus. Furthermore, region-specific molecular signatures create a strong bias intrinsic to astrocytes toward generating neurons of matching regional identity when reprogrammed by the proneural factor Neurog2. This latter finding is in line with reprogramming of cortical astrocytes into neurons with layer-specific properties in vivo (*30*), where a tight lineage relationship between starting and target cell is likely to exist. The transcriptional context of the starting cell might even account for acquisition of specific neuronal fates where region-specific determinants may be expressed more coincidently, such as fibroblast conversion into retinal photoreceptors (*31*).

Despite their common developmental origin, neurons and astrocytes constitute cell types easily distinguishable by their morphological and electrophysiological properties. However, our study reveals that these two cell types show an unexpected overlap in the expression of genes that confer regional identity. Such overlap can be found at the single-cell level and extends into adulthood. Among the shared genes, there was a substantial amount of transcription factors, many of which play well-described roles in neuron subtype specification (e.g., *Gbx2*, *Lef1*, *Fefz2*, and *Tbr1*) (*29*, *32*–*34*). The physiological function in astrocytes of the shared region-specific genes remains to be determined. Future experiments should decipher whether these genes may adopt different functions in astrocytes as compared to neurons or whether their shared expression might act as a code to facilitate region-specific interactions of astrocytes with their sibling neurons (*35*). While these scenarios are not mutually exclusive, the latter may provide an attractive mechanism by which neurons could modulate the spatial distribution of astrocytes (*13*). Our clonal analyses reveal that neurons and sibling astrocytes originating from the same thalamic progenitor clone populate very similar territories, respecting boundaries among thalamic nuclei, extending earlier observations of the existence of nucleus-specific progenitor domains in the thalamus (*26*, *27*, *36*). Recent single-cell spatial transcriptomic mapping has revealed that in the cortex, astrocytes exhibit heterogeneity that does not follow neuronal layering (*13*). However, it is not shown whether neurons and astrocytes populating the same neuronal layer are more likely to be clonally related than those of distinct layers. Nevertheless, cortical regional identity is clearly computed along the anteroposterior and mediolateral axes (*19*), and, indeed, our single-cell data analysis shows that gene expression profiles are shared by astrocytes and neurons along the anteroposterior and mediolateral axes and, thus, it may serve as a mechanism to impart cortical areal identity also to astrocytes, as observed in the thalamus. Most likely, different dimensions of gene expression patterns underlie the unexpected molecular and spatial heterogeneity of astrocytes in the CNS.

It seems plausible that the shared region-specific gene expression is accounted for by epigenetic signatures inherited from a common progenitor and maintained throughout postmitotic development. Our data provide evidence for region-specific differences in the epigenetic state of regulatory elements of these genes in cortical and thalamic astrocytes, even up to the level of thalamic nuclear divisions. Conversely, these region-specific genes apparently escape the long-term epigenetic repression that occurs at neuronal gene loci at the developmental switch from neurogenesis to gliogenesis (*37*, *38*). The epigenetic configuration at region-specific genes might function as a latent mechanism to keep some neuronal expressed genes in a “poised state” in astrocytes, which may become activated by reprogramming factors such as shown here. The fact that epigenetic configurations are heritable through cell divisions (*4*, *38*, *39*) might confer astrocytes with a specific and long-lasting regional differentiation potential as may occur during injury-induced neurogenesis (*40*, *41*). Last, the fine-grained heterogeneity of astrocytes between and within brain regions [this study and (*10*, *13*)] may provide a basis for reconstructing diseased brain circuits that require the generation of multiple neuron types (*30*, *42*), with a minimal number of molecular manipulations.

## MATERIALS AND METHODS

### Mouse strains

All transgenic animals used in this study were maintained on ICR/CD-1, FVB/N-Tg, or C57BL/6J genetic backgrounds and genotyped by PCR. The day of the vaginal plug was stipulated as E0.5. The *Gfap::Gfp* line (*16*) (the Jackson Laboratory, stock number 003257) was in an FVB/N-Tg genetic background, the *Gad67::Gfp* line (*43*) was in C57BL/6J, and the *R26^tdTomato^* Cre-dependent line (the Jackson Laboratory, stock number 007908) and the *Gbx2^CreERT2/+^* mouse line (*18*) were in an ICR/CD-1 genetic background. Tamoxifen induction of Cre recombinase in the double-mutant embryos (*Gbx2^CreERT2/+^*::*R26^tdTomato^*) was performed as previously described (*44*). The Committee on Animal Research at the University Miguel Hernández approved all the animal procedures, which were carried out in compliance with Spanish and European Union regulations.

### Isolation of astrocytes and neurons for RNA-seq

The brains (four brains were pooled for each sample) were extracted in ice-cold KREBS solution and cut in the vibratome in 300-μm slices, and cells were dissociated as in a previous publication (*22*). Thalamic nuclei (dLG, VPM, and MGv) and somatosensory cortex (S1) were dissected and pooled in cold dissociation medium [20 mM glucose, 0.8 mM kynurenic acid, 0.05 mM d,l-2-amino-5-phosphonovaleric acid (APV), penicillin (50 U/ml), streptomycin (0.05 mg/ml), 0.09 M Na_2_SO_4_, 0.03 M K_2_SO_4_, and 0.014 M MgCl_2_]. The tissue was transferred to sterile conditions and enzymatically digested in dissociation medium supplied with l-cysteine (0.16 mg/ml) and 70 U papain (Sigma-Aldrich) set to pH 7.35, at 37°C for 30 min with repeated shaking. The enzyme was then inhibited with dissociation medium containing ovomucoid (0.1 mg/ml) (Sigma-Aldrich) and bovine serum albumin (BSA) (0.1 mg/ml) set to pH 7.35, at room temperature. Tissue was transferred to iced Opti-MEM (Life Technologies) supplied with 20 mM glucose, 0.4 mM kynurenic acid, and 0.025 mM APV and mechanically dissociated until a single-cell suspension was obtained. Cells were concentrated by centrifugation with 850 rpm for 5 min and filtered through a cell strainer (BD Falcon). The genetically labeled live cells were separated based on green or red fluorescence intensity using fluorescence-activated cell sorting (FACSAria III, BD). FACS-purified cells were collected directly in lysis buffer of the RNeasy Micro Kit (Qiagen, no. 74004) that was used to recover total RNA according to the manufacturer’s instructions. RNA quality for all samples was measured on an Agilent Bioanalyzer 2100 system. All samples with RNA Integrity Number (RIN) > 7 were used as input to library construction.

### Library preparation and RNA-seq

Library construction and sequencing were performed at the CNAG-CRG (Centro Nacional de Análisis Genómico) genomics core facility (Barcelona, Spain). Briefly, cDNA multiplex libraries were prepared using SMARTer Ultra Low RNA Kit v4 (Takara, no. 634894) and NEBNext Ultra DNA Library Prep for Illumina according to the manufacturer’s instructions (NEB, no. E7645). Libraries were sequenced together in a single flow cell on an Illumina HiSeq 2500 platform using v4 chemistry in 1 × 50 bp (base pair) single-end mode. A minimum of 25 million reads were generated from each library.

### Bioinformatic analysis of the RNA-seq

RNA-seq analyses were performed as previously described (*45*) with minor modifications: Quality control of the raw data was performed with FastQC (v0.11.7) and sequenced dataset adapters were trimmed using Cutadapt (v2.3) and Trim Galore (v0.6.1). RNA-seq reads were mapped to the mouse genome (GRCm.38.p6/mm10) using STAR (v2.7.0d), and SAM/BAM files were further processed using SAMtools (v1.9). Aligned reads were counted and assigned to genes using Ensembl release 95 gene annotation and FeatureCounts, Subread (v1.6.4) (*46*). Normalization of read counts and differential expression analyses were performed using DESeq2 (v1.22.2) and Bioconductor (v3.8) in the R statistical computing and graphics platform (v3.5.1 “Feather Spray”).

In the analysis datasets of cortical astrocytes and thalamic astrocytes and neurons generated for this study, significantly DEGs were identified using statistical significance threshold [Benjamini-Hochberg (BH)–adjusted *P* value < 0.1] and absolute log_2_ fold change (log_2_FC) > 0 using shrunken log_2_FC using the adaptive *t* prior Bayesian shrinkage estimator “apeglm” (tables S1, S3, and S4) (*47*). To identify the top most differentially enriched genes between cortical and thalamic neurons, we used data generated in this study for thalamic neurons (P0) and publicly available dataset for cortical neurons (P1) from a previously published study (GSE63482) (*20*). Datasets from (*20*) consist of RNA-seq profiles of multiple classes of FACS-purified cortical neurons from ICR/CD-1 mice: callosal projecting neurons (CPN, *n* = 2), corticothalamic projecting neurons (CThPN, *n* = 2), and subcerebral projecting neurons (ScPN, *n* = 2) (*20*). Neuronal datasets from the cortex and thalamus were aligned from the raw sequence, and gene counts were generated using the same pipeline as indicated previously. Gene counts were normalized using the median of ratios method in DESeq2 R package, and the ratio between gene counts (regularized logarithm transformation of the normalized counts) were used to identify the top 400 most differentially enriched genes between cortical and thalamic neurons. Hypergeometric test (one-sided Fisher’s exact test) was performed to test independence between lists of enriched or significantly DEGs from neurons and astrocytes from different brain regions and to obtain estimated odds ratios. RNA-seq coverage tracks for selected genes were generated using Integrative Genomics Viewer (IGV) (v2.4.14) and plotted in a 5′ to 3′ direction. Hierarchical clustering analysis was performed using “Manhattan” distance and “Ward.2” clustering method metrics to visualize significantly up-regulated and down-regulated genes. In the functional enrichment analysis of the datasets from astrocytes, a more restrictive filtering criterion was used to detect high significantly DEG based on simultaneous threshold of BH-adjusted *P* value < 0.1 and absolute log_2_FC > 0.322. This analysis revealed 508 versus 444 DEGs enriched in the thalamus and cortex, respectively. The GO overrepresentation analysis and GSEA were performed using clusterProfiler (v3.10.1) (*48*). All enriched terms were considered significant at adjusted *P* values by BH with *P* value cutoff < 0.01 and 0.1, in the GO overrepresentation analysis and GSEA, respectively. The reference gene set used to perform the analysis was C5 (GO Biological Process) collection from the Molecular Signatures Database (MSigDB) (v6.2).

### Bioinformatic analysis of the scRNA-seq

We analyzed recent work from scRNA-seq to interrogate thalamic and cortical cellular heterogeneity (*23*). The sequence data are publicly available at the National Center for Biotechnology Information (NCBI) Sequence Read Archive (SRA) under accession SRP135960 (*23*). Briefly, scRNA-seq datasets (postfiltered count matrices) for the thalamus and cortex were downloaded from the associated wiki and processed with Seurat R package (v3.1.4) (*49*). First, we performed quality control analysis that confirmed that the data were of high quality. All cells had more than 600 detected molecules (UMIs) and the proportion of mitochondrial reads was below 5% for the vast majority of cells (see fig. S2, A and B). Next, data were preprocessed (log normalization and scaling) before performing linear dimensional reduction (PCA). Graph-based clustering approach using the top 30 principal components was used to identify cell populations (resolution was fixed to 0.8). FindAllMarkers function with default parameters was used to identify gene markers for each cluster and to assign cell-type identity to clusters (see fig. S2, C and D).

Cortical and thalamic scRNA-seq datasets were subsequently integrated as previously described (*50*). The UMAP (Uniform Manifold Approximation and Projection) algorithm was used to nonlinear dimensionality reduction, visualization, and exploratory analysis of the datasets. Differential expression analyses between thalamic and cortical neurons and astrocytes were performed using the FindMarkers function based on the nonparametric Wilcoxon rank sum test with the following parameters (logFC.threshold = 0.1; min.pct = 0). Genes with BH-adjusted *P* value < 0.1 were considered significantly differentially expressed (tables S2 and S5).

### In utero electroporation of StarTrack vectors

For in utero electroporation, a procedure previously described was followed (*51*). Pregnant females (E11.5) were deeply anesthetized with isoflurane to perform laparotomies. The embryos were exposed, and the third ventricles of the embryonic brains were visualized through the uterus with an optic fiber light source. The combination of the plasmids of the StarTrack method at a final concentration of 2 μg/μl was mixed with 0.1% Fast Green (Sigma-Aldrich), as previously described (*12*, *25*). The plasmids used consisted of the coding sequence of six fluorescent proteins (EGFP, mCherry, mKusabian Orange, mTSapphire, mCerulean, and EYFP) subcloned under the regulation of the GFAP or UbC promoters for targeting specifically the astrocytes or all the cell types. Each reporter gene could be directed to the cytoplasm (PB-GFAP/UbC-XFP) or to the nucleus of the cell by fusing it with the H2B histone protein (PB-GFAP/UbC-H2B-XFP). Constructs were flanked by PiggyBac sequences to be inserted into the genome of the targeted cell by a PiggyBac transposase. The plasmids were injected into the third cerebral ventricle by an injector (Nanoliter 2010, WPI). For electroporation, five square electric pulses of 45 V and 50 ms were delivered through the uterus at 950-ms intervals using a square pulse electroporator (CUY21 Edit, NepaGene Co., Japan). The surgical incision was then closed, and embryos were allowed to develop until P8. In the electroporated animals with the UbC-StarTrack combination, tamoxifen was administered at P1 as previously described (*25*) for removing nonintegrated copies of the electroporated plasmids through the Cre recombinase system.

### Measurement of thalamic astrocytic clones

Images were acquired with an Olympus FV1000 confocal IX81 microscope/FV10-ASW software following previously defined settings (*12*). All the pictures were acquired with a 20× oil immersion objective and analyzed with ImageJ software. Only electroporated animals with labeled cells in the three first order thalamic nuclei (dLG, VPM, and MGv) were used. Then, only clones with at least three cells and with the presence of more than one reporter were analyzed.

First, we assigned a binary code to every cell based on the presence or absence of each reporter protein in the cytoplasm and/or the cellular nuclei and the expression of the neuronal marker NeuN in order to distinguish neurons from glial cells. Once all the cells had been analyzed, they were grouped on the basis of their shared binary code, thereby identifying those cells that originated from the same progenitor. Then, we quantified the distribution (in %) of cells belonging to the same clone across the thalamic nuclei.

### Virus production

For the production of the retrovirus, Lenti-X 293T cells (catalog no. 632180, Clontech) were plated on 5- to 10-cm dishes. Encapsulation plasmids containing gag-pol and vsv-g sequences (provided by V. Borrell) were cotransfected with the plasmid of interest using LipoD293 (catalog no. SL100668, SignaGen). The medium was changed after 5 hours, and the virus was collected after 72 hours using Lenti-X concentrator (catalog no. 631231, Clontech).

### In vivo viral and BrdU injections

Pups at P3 were anesthetized on ice and placed in a digital stereotaxic. The virus was injected using an injector (Nanoliter 2010, WPI) in the thalamus or cortex through a small skull incision. BrdU was injected intraperitoneally at 50 mg/kg immediately after viral injections from stock solution (10 mg/ml).

### Astrocyte primary cultures

Postnatal astroglia was cultured as previously described (*52*). Briefly, after removal of the meninges, the cortices (somatosensory and visual) and the thalamus from P4 to P6 mice were dissected and dissociated mechanically in cold KREBS 1×. Subsequently, cells were centrifuged for 10 min at 1000 rpm, resuspended, and plated in a medium consisting of DMEM/F12 (Gibco), 3.5 mM glucose (Sigma-Aldrich), 10% fetal calf serum (Gibco), 5% horse serum (Gibco), 1× GlutaMAX (Fisher), and antibiotic/antimycotic (100 U/μl) (Fisher) and supplemented with B27 2% (Gibco), epidermal growth factor (10 ng/ml) (EGF; Roche), and fibroblast growth factor 2 (10 ng/ml) (FGF2; Roche). Oligodendrocyte precursor cells were removed by brusquely shaking the culture flasks several times when changing the medium after 2 or 3 days. Cells were passaged after 1 week using trypsin/EDTA (Gibco) and plated on poly-d-lysine (Sigma-Aldrich) glass-coated coverslips at a density of 50,000 to 70,000 cells per coverslip (in 24-well plates; BD Biosciences) in the same medium. The vast majority of the cells (>90%) were positive for glial fibrillary acidic protein (Gfap). Nuclei-specific thalamic astrocytic cultures were performed similarly but with a few modifications. Brains were dissected out and cut in a vibratome in 300-μm slices in cold KREBS to dissect the three principal sensory thalamic nuclei: dLG nucleus, the somatosensory VPM nucleus, and the auditory MGv. Thalamic nuclei were then mechanically dissociated and plated on six-well plates and passed when confluent. Astrocytes were infected with CAG-*GFP*-IRES-*GFP*, CAG*-(Flag)Neurog2*-IRES-*DsRed*, CAG*-(Flag)Neurog2*-IRES-*TauGFP*, or CAG*-Gbx2-*IRES*-DsRed* retroviruses. After 24 hours, the medium was changed by a differentiation medium containing DMEM/F12 (Gibco), 3.5 mM glucose (Sigma-Aldrich), 1× GlutaMAX (Fisher), and antibiotic/antimycotic (100 U/μl) (Fisher) and supplemented with B27 2% (Gibco). BDNF (Sigma-Aldrich) was added at 20 ng/ml every fourth day during the differentiation process.

### Histology

For immunofluorescence of reprogrammed neurons in vitro, cultures were fixed with 4% paraformaldehyde (PFA) in phosphate-buffered saline (PBS) (0.01 M) for 10 to 15 min at room temperature. Cultures were first incubated for 1 hour at room temperature in a blocking solution containing 2% BSA (Sigma-Aldrich) and 0.15% Triton X-100 (Sigma-Aldrich) in 0.01 M PBS. Subsequently, the cells were incubated overnight at 4°C with the primary antibodies listed in table S6. The cells were then rinsed in 0.01 M PBS and incubated for 2 hours at room temperature with adequate secondary antibodies listed in table S6. Counterstaining was performed by the fluorescent nuclear dye 4′,6-diamidino-2-phenylindole (DAPI) (Sigma-Aldrich).

For histology in postnatal brains, mice were perfused transcardially first with 0.01 M PBS and 4% PFA. Brains were kept on 4% PFA overnight, embedded with 3% agarose in 0.01 M PBS, and cut into slices of 80 μm of thickness in a vibratome (Leica). For Tbr1, Ctip2, Aldh1l1, Rorα, and Lef1 antibodies, an antigen retrieval step with sodium citrate was performed. For BrdU detection, slices were first incubated with 2 N HCl and 0.5% Triton X-100 at 37°C for 30 min, followed by an incubation with borax buffer at room temperature. Slices were incubated for 1 hour at room temperature in a blocking solution containing 1% BSA, 2% donkey serum, 2% goat serum, and 0.4% Triton X-100 in 0.01 M PBS and subsequently incubated overnight at 4°C with primary antibodies. Slices were incubated for 2 hours at room temperature with the appropriate secondary antibodies, washed, incubated with DAPI, and mounted. Images were acquired with a Leica DFC550 camera into a Leica DM5000B microscope, with an Olympus FV1000 confocal IX81 microscope/FV10-ASW software, or with a Zeiss confocal LSM880.

### Fluorescence in situ hybridization

*Gfap::Gfp* brains were cut into slices of 100 μm of thickness in a vibratome (Leica). Slices were dehydrated, incubated for 15 min with 2% H_2_O_2_ in EtOH at room temperature for blocking endogenous peroxidase, and rehydrated. Then, slices were washed first with PBS and 0.1% Tween 20 (PBT), then with a detergent mix [1% NP-40, 1% SDS, 0.5% sodium deoxycholate, 50 mM tris-HCl (pH 8), 1 mM EDTA, and 150 mM NaCl] three times for 20 min, and postfixed with 4% PFA. After three washes with PBT, slices were incubated with prehybridization solution [50% deionized formamide, 5× SSC (pH 5.3), heparin (50 μg/ml), tRNA (50 μg/ml), single-stranded DNA (50 μg/ml), and 0.1% Tween 20] for 1 hour at 65°C in a humid chamber and then incubated overnight with the corresponding probe in prehybridization solution at 65°C.

The next day, slices were washed four times with prewarmed washing solution [50% formamide, 2× SSC (pH 5.3), and 1% SDS] at 65°C and four times with MABT [100 mM maleic acid, 150 mM NaCl, 0.19 M NaOH (pH 7.5), and 0.1% Tween 20]. Slices were then incubated with blocking solution [2% Blocking Reagent (Sigma-Aldrich, no. 11096176001) in MABT] for 2 hours and then incubated overnight at 4°C with anti–digoxigenin-POD (Sigma-Aldrich, no. 11207733910) diluted 1/500 in blocking solution.

Slices were washed four times with MABT and then revealed with TSA PLUS CYANINE 3 (Akoya, SKU NEL744001KT) diluted 1/500 in MABT. Once revealed, slices were washed with MABT and then immunofluorescence was performed as described above.

### Purification of total RNA and quantitative real-time PCR

For specific isolation of reprogrammed astrocytes, a previously published method was followed (*20*) but with some modifications for cultured cells. Astrocytes from the thalamus, cortex, dLG, VPM, and MGv were cultured and infected with *Neurog2* retrovirus, and after 10 days in vitro, they were collected by applying trypsin/EDTA (Gibco) to the plate, resuspended with culture medium, and centrifuged. Reprogrammed astrocytes were fixed with PFA 1% for 10 min at 4°C, after which the PFA was quenched by adding 55 μl of glycine, 1.25 M per 500 μl of PFA solution. Immunocytochemistry against Tuj1 and RFP was performed, and cells were separated (Tuj1^+^/RFP^+^ versus Tuj1^−^/RFP^+^) by a flow cytometer (BD FACSAria) based on their fluorescence (see schema on fig. S8, C to E). Once the cells were collected, they were centrifuged and incubated for 3 hours at 50°C with lysis buffer, their RNA was purified using TRIzol (Fisher), and cells were resuspended in RNase-free water.

cDNA was obtained from 1 μg of total RNA using the specific protocol for first-strand cDNA synthesis in two-step reverse transcription (RT)–PCR using the High-Capacity cDNA Reverse Transcription Kit (Fisher) and stored at −20°C. qPCR was performed in a StepOnePlus real-time PCR system (Applied Biosystems, Foster City, CA, USA) using the MicroAmp fast 96-well reaction plate (Applied Biosystems) and the Power SYBR Green PCR Master Mix (Applied Biosystems). The primers used for detecting the expression of the different genes are listed in table S7. A master mix was prepared for each primer set containing the appropriate volume of SYBR Green, primers, and template cDNA. All reactions were performed in triplicate. The amplification efficiency for each primer pair and the cycle threshold (Ct) were determined automatically by the StepOne Software, v2.2.2 (Applied Biosystems). Transcript levels were represented relative to the *Gapdh* signal, adjusting for the variability in cDNA library preparation.

### Patch-clamp recordings of iNs

For the electrophysiological analysis, astrocytes were infected with a retrovirus encoding CAG-*Neurog2*-ires-*TauGFP*. After 3 weeks, cultures were transferred to the recording chamber and were perfused with standard artificial cerebrospinal fluid (aCSF) containing the following: 119 mM NaCl, 5 mM KCl, 1.3 mM MgSO_4_, 2.4 mM CaCl_2_, 1 mM NaH_2_PO_4_, 26 mM Na_2_HCO_3_, and 11 mM glucose. The aCSF was perfused at a rate of 2.7 ml min^−1^, continuously bubbled with a gas mixture of 95% O_2_ + 5% CO_2_, and warmed at 30° to 32°C.

Somatic whole-cell recordings were made under visual control using an upright microscope (Leica DM-LFSA) and a water immersion (20 or 40×) objective. The intracellular solution contained the following: 130 mM K-gluconate, 5 mM KCl, 5 mM NaCl, 0.2 mM EGTA, 10 mM Hepes, 4 mM Mg-ATP, and 0.4 mM Na-GTP, pH 7.2 adjusted with KOH; 285 to 295 mOsm. Recordings were obtained in current-clamp and/or voltage-clamp mode with a patch-clamp amplifier (MultiClamp 700A, Molecular Devices, USA). No correction was made for the pipette junction potential. Voltage and current signals were filtered at 2 to 4 kHz and digitized at 20 kHz with a 16-bit resolution analog to digital converter (Digidata 1550B, Axon Instruments). The generation and acquisition of pulses were controlled by pClamp 10.6 software (Axon Instruments). Patch pipettes were made from borosilicate glass [1.5 mm OD (outer diameter), 0.86 mm ID (inner diameter), with inner filament] and had a resistance of 4 to 7 megohms when filled. Neurons in which series resistance was >30 megohms were discarded. Quantification of intrinsic membrane properties and spontaneous neuronal activity was performed on Clampfit 10.7 (Axon Instruments). The presence of putative spontaneous excitatory postsynaptic currents (sEPSCs) was assessed in voltage clamp recordings at −70 mV.

### In silico *Neurog2* binding sites determination

In silico analysis was performed to find out Neurog2 binding sites across the whole genome using FIMO Motif Scanning from MEME Suite (v5.0.2) (*53*). Neurog2 transcription factor motif (NGN2_MOUSE.H11MO.0.C) from HOCOMOCO database (v11) and mouse genome (GRCm38.p6 GenCode M18) were used to carry out this analysis. Neurog2 binding sites were annotated to genes using ChIPseeker (v1.18) (*54*) and Bioconductor (v3.8) in the R statistical computing and graphics platform (v3.5.1 Feather Spray). We retrieved genomic regions and selected binding sites [promoters, 5′UTR (5′ untranslated region), first intron and first exon] whose location was ±3 kb of GENCODE annotated TSSs (transcription start sites) of protein-coding genes. These criteria retrieved 180,611 putative Neurog2 binding sites belonging to 20,478 protein coding genes. Chromatin immunoprecipitation sequencing (ChIP-seq) coverage tracks for selected genes were generated using IGV (v2.4.14) and plotted in a 5′ to 3′ direction based on publicly available datasets from forebrain samples of H3K4me3 (ENCSR258YWW experiment) and H3K27me3 (ENCSR070MOK experiment) histone marks at P0 extracted from the ENCODE Project (see fig. S9).

### ChIP for H3K4me3 and H3K27me3

ChIP assays were performed following a previously published protocol (*55*). Cultured astrocytes from the thalamus and cortex were collected after 1 week in vitro when confluence is reached, centrifuged, and resuspended to approximately 500,000 cells. Cells were fixed with 1% PFA in PBS for 10 min at room temperature and quenched with 55 μl of glycine, 1.25 M per 500 μl of PFA solution with orbital shaking. After that, cells were lysed in 300 μl of SDS lysis buffer (0.5% SDS, 10 mM EDTA, and 50 mM tris-HCl) supplemented with protease inhibitor cocktail (Roche, 11836153001), sonicated for 10 min in a Diagenode Bioruptor Pico, precleared with 30 μl of washed Dynabeads (Invitrogen, 10003D), and diluted five times in ChIP IP buffer [20 mM Hepes, 0.2 M NaCl, 2 mM EDTA, 0.1% Na-DOC, 1% Triton X-100, and BSA (5 mg/ml)]. One percent of each sample was kept as input. Samples were divided into three tubes and incubated overnight at 4°C in a rotating wheel with 2.5 μg per tube of the anti-H3K4me3 (Sigma-Aldrich, 07-473), anti-H3K27me3 (Abcam, ab6002), or control IgG antibody. The next day, washed and saturated Dynabeads were added and incubated with the samples for 2 hours at 4°C. Dynabeads were washed five times with LiCl buffer (50 mM Hepes, 1 mM EDTA, 1% NP-40, 1% Na-DOC, and 0.5 M LiCl) and once with TE buffer (10 mM Tris-HCl and 1 mM EDTA). Antibody/chromatin complexes together with the inputs were eluted by adding 100 μl of elution buffer (50 mM NaHCO_3_ and 1% SDS), 10 μl of NaCl (5 M), and 1 μl of proteinase K (Sigma-Aldrich, 3115836001) to each tube and put on a thermomixer, shaking at 1000 rpm at least 2 hours at 60°C. Samples and inputs were decross-linked by heating for 15 min at 95°C. Both samples and inputs were treated with RNase A (Roche, 10109142001) for 30 min at 65°C, and the DNA was purified with phenol/chloroform and ethanol-precipitated. Primers used for detecting the immunoprecipitated genomic regions are listed in table S7.

### Primer design

For RNA expression analysis, Primer3 and Blast tools from NCBI webpage were used, using the accession numbers of the coding sequences of the genes of interest. For ChIP experiments, we used the information obtained from the in silico *Neurog2* binding sites analysis and the open-source information of the ENCODE project. For primers design, regions on the promoters of candidate genes that included a putative binding site for *Neurog2* and that were enriched in H3K4me3 and H3K27me3 signal were selected.

### Quantification and statistical analysis

Statistical analysis was carried out in GraphPad Prism (v.6), Origin (v.8.0), and R (v3.5.1 Feather Spray) statistical computing and graphics platform. Data are presented as means ± SEM or with box-and-whisker plots, which give the median, 25th and 75th percentiles, and range. Statistical comparison between groups was performed using paired or unpaired two-tailed Student’s *t* test or Mann-Whitney *U* test nonparametric two-tailed test when data failed a Kolmogorov-Smirnov or a Shapiro-Wilk normality test. For multiple comparison analysis, a one-way analysis of variance (ANOVA) test with Holm-Sidak’s multiple comparisons test was used, and Kruskal-Wallis test with Dunn’s multiple comparisons test was used when data failed a Kolmogorov-Smirnov or a Shapiro-Wilk normality test. Simple effect analysis was performed when interaction was significant. *P* values < 0.05 were considered statistically significant and set as follows: *P < 0.05, **P < 0.005, and ***P < 0.0005. In the bioinformatical analysis, DEGs were identified using a statistical significance threshold (BH-adjusted *P* value < 0.1) and set as follows: *adj. *P* < 0.1, **adj. *P* < 0.01, and ***adj. *P* < 0.001. No statistical methods were used to predetermine the sample size, but our sample sizes are considered adequate for the experiments and consistent with the literature. The mice were not randomized. The investigators were blinded to sample identity.

For Fig. 1B, PCA of astrocytes shows only the first two principal components, PC1 represents 59% variance and PC2 represents 15% variance (*n* = 4 Ctx, *n* = 4 to 5 each Th nucleus). For Fig. 1C, DE analysis (adj. *P* < 0.1, log_2_FC > 0; 1675 DEG As-Th versus 1287 DEG As-Ctx). For Fig. 1D, DE analysis (adj. *P* < 0.1, log_2_FC > 0; 1675 DEG As-Th versus 1287 DEG As-Ctx) (table S1). For Fig. 1E, GSEA: thalamus development (GO:0021794) (NES = 1.666; *P* = 0.028; adj. *P* = 0.074), diencephalon development (GO:00221536) (NES = 1.889; *P* = 0.018; adj. *P* = 0.052), cerebral cortex neuron differentiation (GO:0021895) (NES = −2.119; *P* = 0.002; adj. *P* = 0.011), and telencephalon regionalization (GO:0021978) (NES = −1.879; *P* = 0.008; adj. *P* = 0.029) produced with a more restrictive DE analysis (adj. *P* < 0.1, log_2_FC > 0.322, As-Th: 508 DEGs, As-Ctx: 444 DEGs). Figure 1F used a more restrictive DE analysis (adj. *P* < 0.1, log_2_FC > 0.322, As-Th: 508 DEGs, As-Ctx: 444 DEGs).

For Fig. 2B, hypergeometric test (one-sided Fisher’s exact test). As-Th enriched in Ns-Th ***P = 3.649224 × 10^−37^, OD = 4.7272; As-Th enriched in Ns-Ctx ns *P =* 0.9981097, OD = 0.5754116; As-Ctx enriched in Ns-Ctx ***P = 3.304775 × 10^−11^, OD = 2.669471, As-Ctx enriched in Ns-Th ns *P =* 0.9957097, OD = 0.5668416. Quantification was recovered from data of RNA-seq analysis of astrocytic DEGs (adj. *P* < 0.1, log_2_FC > 0; 1675 DEG As-Th versus 1287 DEG As-Ctx) and top 400 neuronal genes. For Fig. 2E, DE analysis of astrocytes, Wilcoxon rank sum test (adj. *P* < 0.1, log_2_FC > 0.1, As-Th: 549 DEGs, As-Ctx: 1106 DEGs). For the DE analysis of neurons, Wilcoxon rank sum test (adj. *P* < 0.1, log_2_FC > 0.1, Ns-Th: 2425 DEGs, Ns-Ctx: 1845 DEGs) (table S2). For Fig. 2F, hypergeometric test (one-sided Fisher’s exact test). As-Th enriched in Ns-Th ***P = 4.001415 × 10^−73^, OD = 5.57147; As-Th enriched in Ns-Ctx ns *P =* 0.9992975, OD = 0.6456831; As-Ctx enriched in Ns-Ctx ***P = 2.066775 × 10^−159^, OD = 7.142444; and As-Ctx enriched in Ns-Th ns *P =* 0.9962708, OD = 0.7517044. Quantification was recovered from the data of scRNA-seq analysis of astrocytic DEGs (adj. *P* < 0.1, log_2_FC > 0.1, As-Th: 549 DEGs, As-Ctx: 1106 DEGs) and neuronal DEGs (adj. *P* < 0.1, log_2_FC > 0.1, Ns-Th: 2425 DEGs, Ns-Ctx: 1845 DEGs) (table S2).

For Fig. 3B, PCA shows only the first two principal components in astrocytes of the three thalamic nuclei, PC1 represents 41% variance and PC2 represents 24% variance (*n* = 4 to 5 replicates each; table S3). For Fig. 3C, PCA shows only the first two principal components, PC1 represents 47% variance and PC2 represents 32% variance (*n* = 3 to 4 replicates each; table S4). For Fig. 3D, hypergeometric test (one-sided Fisher’s exact test) from intersect between the populations of genes for the comparison of significant overexpression in As-dLG and enriched in Ns-dLG ***P = 1.750051 × 10^−11^, OD = 4.4.292546; significant overexpression in As-MGv and enriched in Ns-dLG ns *P =* 0.9956851, OD = 0.3838985; significant overexpression in As-VPM and enriched in Ns-dLG ns *P =* 0.9999685, OD = 0.5766173; significant overexpression in As-dLG and enriched in Ns-MGv ns *P =* 0.7531583, OD = 0.8598092; significant overexpression in As-MGv and enriched in Ns-MGv ***P = 3.358423 × 10^−18^, OD = 3.946944; significant overexpression in As-VPM and enriched in Ns-MGv ns *P =* 1, OD = 0.3334283; significant overexpression in As-dLG and enriched in Ns-VPM ns *P =* 0.8043838, OD = 0.7983417; significant overexpression in As-MGv and enriched in Ns-VPM ns *P =* 0.3478912, OD = 1.123736; significant overexpression in As-VPM and enriched in Ns-VPM ***P = 1.256227 × 10^−25^, OD = 2.495969. Quantification was recovered from the data of RNA-seq analysis of astrocytic DEGs (adj. *P* < 0.1, log_2_FC > 0; 221 DEG As-dLG, 1771 DEG As-VPM, and 278 DEG As-MGv) (table S3) and neuronal DEGs (adj. *P* < 0.1, log_2_FC > 0; 705 DEG Ns-dLG, 961 DEG Ns-VPM, 1330 DEG Ns-MGv) between distinct sensory-modality thalamic nuclei (table S4).

For Fig. 4C, Kruskal-Wallis test, with Dunn’s multiple comparisons test. *n* = 5 electroporated mice. For dLG clones, *n* = 59 clones: ***P < 0.0001; dLG [confidence interval (CI): 77.24 to 87.74%] versus VPM (CI: 6.932 to 15.36%), ***P < 0.0001; dLG versus MGv (CI: 2.909 to 9.824%), ***P < 0.0001; VPM versus MGv, ns *P* = 0.7795. For VPM clones, *n* = 179 clones: ***P < 0.0001; dLG (CI: 3.65 to 7.266%) versus VPM (CI: 84.69 to 90.01%), ***P < 0.0001; VPM versus MGv (CI: 5.131 to 9.401%), ***P < 0.0001; dLG versus MGv, ns *P* > 0.9999. For MGv clones, *n* = 82 clones: ***P < 0.0001; dLG (CI: 0.7806 to 4.859%) versus MGv (CI: 78.39 to 88.03%), ***P < 0.0001; VPM (CI: 8.903 to 16.60%) versus MGv, ***P < 0.0001; dLG versus VPM, *P = 0.0253. For Fig. 4G, Kruskal-Wallis test, with Dunn’s multiple comparisons test. *n* = 4 electroporated animals. For dLG mixed clones, *n* = 52 clones. For all cells, ***P < 0.0001; dLG (CI: 72.52 to 81.64%) versus VPM (CI: 13.12 to 21.29%), ***P < 0.0001; dLG versus MGv (CI: 3.346 to 7.865%), ***P < 0.0001. For neurons, ***P < 0.0001; dLG (CI: 64.72 to 82.4%) versus VPM (CI: 11.40 to 27.25%), ***P < 0.0001; dLG versus MGv (CI: 3.305 to 10.61%), ***P < 0.0001. For no neurons, ***P < 0.0001; dLG (CI: 57.50 to 78.95%) versus VPM (CI: 14.45 to 33.54%), ***P < 0.0001; dLG versus MGv (CI: 1.136 to 14.42%), ***P < 0.0001. For VPM clones, *n* = 71 clones. For all cells, ***P < 0.0001; dLG (CI: 9.353 to 16.29%) versus VPM (CI: 74.28 to 82.26%), ***P < 0.0001; VPM versus MGv (CI: 5.639 to 11.73%), ***P < 0.0001. For neurons, ***P < 0.0001; dLG (CI: 17.88 to 33.37%) versus VPM (CI: 54.55 to 71.69%), ***P < 0.0001; VPM versus MGv (CI: 4.817 to 17.69%), ***P < 0.0001. For no neurons, ***P < 0.0001; dLG (CI: 0.7925 to 7.465%) versus VPM (CI: 74.88 to 90.40%), ***P < 0.0001; VPM versus MGv (CI: 5.491 to 19.11%), ***P < 0.0001. For MGv clones, *n* = 7 clones. For all cells, ***P < 0.0001; VPM (CI: 6.127 to 33.87%) versus MGv (CI: 44.95 to 87.91%), **P = 0.0099; dLG (CI: 1.42 to 25.72%) versus MGv, **P = 0.0011. For neurons, ns *P* = 0.7463; VPM (CI: −2.264 to 68.93%) versus MGv (CI: 0.3102 to 85.40%), ns *P* > 0.9999; dLG (CI: −10.49 to 58.11%) versus MGv, ns *P* = 0.8395. For no neurons, *P = 0.0167; VPM (CI: −7.477 to 28.91%) versus MGv (CI: 35.05 to 107.8%), *P = 0.0209; dLG (CI: −16.73 to 52.45%) versus MGv, *P = 0.0354.

For Fig. 5C, Mann-Whitney *U* test nonparametric two-tailed test. For Lef1, **P = 0.0065, *n* = 6 mice (265 iNs in Th and 103 iNs in Ctx); for Rorα, *P = 0.0286, *n* = 4 mice (69 iNs in Th and 176 iNs in Ctx); for Tbr1, **P = 0.0022, *n* = 5 to 6 mice (202 iNs in Th and 109 iNs in Ctx); and for Ctip2, **P = 0.0022, *n* = 6 mice (202 iNs in Th and 109 iNs in Ctx). For Fig. 5F, ordinary one-way ANOVA and Holm-Sidak’s multiple comparisons test for Rorα [*F* = 13.00, degrees of freedom (df) = 29, ***P < 0.0001], Tbr1 (*F* = 23.56, df = 31, ***P < 0.0001), and Ctip2 (*F* = 30.70, df = 28, ***P < 0.0001), and Kruskal-Wallis test with Dunn’s multiple comparisons test for vGlut2 (***P < 0.0001). For Rorα, Inf. As-Th versus Inf. As-Ctx, ***P < 0.0001, *n* = 6 independent cultures; Inf. As-Th + As-Ctx versus Inf. As-Ctx + As-Th, ***P = 0.0008, *n* = 6; Inf. As-Th + Ns-Ctx versus Inf. As-Ctx + Ns-Th, **P = 0.0022, *n* = 6. For Tbr1, Inf. As-Th versus Inf. As-Ctx, ***P < 0.0001, *n* = 6; Inf. As-Th + As-Ctx versus Inf. As-Ctx + As-Th, ***P < 0.0001, *n* = 6; Inf. As-Th + Ns-Ctx versus Inf. As-Ctx + Ns-Th, ***P < 0.0001, *n* = 6. For Ctip2, Inf. As-Th versus Inf. As-Ctx, ***P < 0.0001, *n* = 6; Inf. As-Th + As-Ctx versus Inf. As-Ctx + As-Th, ***P < 0.0001, *n* = 6; Inf. As-Th + Ns-Ctx versus Inf. As-Ctx + Ns-Th, ***P < 0.0001, *n* = 6. For vGlut2, Inf. As-Th versus Inf. As-Ctx, ***P = 0.0003, *n* = 6; Inf. As-Th + As-Ctx versus Inf. As-Ctx + As-Th, *P = 0.0437, *n* = 7; Inf. As-Th + Ns-Ctx versus Inf. As-Ctx + Ns-Th, *P = 0.0239, *n* = 5. For Fig. 5H, ordinary one-way ANOVA test with Holm-Sidak’s multiple comparisons test. *Sp9*, *F* = 8.924, df = 32, ***P = 0.0008; dLG versus VPM, ***P = 0.0007, *n* = 12 independent cultures; dLG versus MGv, **P = 0.0034, *n* = 12. *Hs6st2*, *F* = 5.128, df = 25, *P = 0.0136; dLG versus VPM, **P = 0.0093, *n* = 10; dLG versus MGv, *P = 0.0331, *n* = 10. *Crabp2*, *F* = 4.702, df = 24, *P = 0.0189; MGv versus dLG, *P = 0.0341, *n* = 10; MGv versus VPM, *P = 0.0147, *n* = 10. *Tshz1*, *F* = 10.97, df = 37, ***P = 0.0002; MGv versus dLG, ***P = 0.0006, *n* = 14; MGv versus VPM, ***P = 0.0003, *n* = 14. *Cck*, *F* = 5.409, df = 30, **P = 0.0099; VPM versus dLG, **P = 0.0064, *n* = 12; VPM versus MGv, *P = 0.0393, *n* = 12.

For Fig. 6B, ordinary one-way ANOVA test with Holm-Sidak’s multiple comparisons test; for *Gbx2* in Th, *F* = 39.71, ***P < 0.0001; Th basal (*n* = 19) versus Th + Neurog2 (*n* = 14), ns *P* = 0.9579, *t* = 0.05318, df = 36; Th basal versus Th + Neurog2 + Gbx2 (*n* = 6), ***P < 0.0001, *t* = 8.429, df = 36; Th + Neurog2 versus Th + Neurog2 + Gbx2, ***P < 0.0001, *t* = 8.128, df = 36. For Ctx, *F* = 167.4, ***P < 0.0001; Ctx basal (*n* = 14) versus Ctx + Neurog2 (*n* = 14), ns *P* = 0.9831, *t* = 0.02134, df = 31; Ctx basal versus Ctx + Neurog2 + Gbx2 (*n* = 6), ***P < 0.0001, *t* = 16.88, df = 31; Ctx + Neurog2 versus Ctx + Neurog2 + Gbx2, ***P < 0.0001, *t* = 16.86, df = 31. *Pou2f2*, for Th, *F* = 20.15, ***P < 0.0001; Th basal (*n* = 12) versus Th + Neurog2 (*n* = 14), *P = 0.0386, *t* = 2.163, df = 30; Th basal versus Th + Neurog2 + Gbx2 (*n* = 7), ***P < 0.0001, *t* = 6.307, df = 30; Th + Neurog2 versus Th + Neurog2 + Gbx2, ***P = 0.0001, *t* = 4.642, df = 30. For Ctx, *F* = 11.79, ***P = 0.0001; Ctx basal (*n* = 14) versus Ctx + Neurog2 (*n* = 12), ns *P* = 0.6091, *t* = 0.5164, df = 32; Ctx basal versus Ctx + Neurog2 + Gbx2 (*n* = 9), ***P = 0.0002, *t* = 4.589, df = 32; Ctx + Neurog2 versus Ctx + Neurog2 + Gbx2, ***P = 0.0007, *t* = 3.986, df = 32. *Tbr1*, for Th, *F* = 0.2125, ns *P* = 0.8095; Th basal (*n* = 20) versus Th + Neurog2 (*n* = 14), ns *P* = 0.8900, *t* = 0.6478, df = 39; Th basal versus Th + Neurog2 + Gbx2 (*n* = 8), ns *P* = 0.9203, *t* = 0.1537, df = 39; Th + Neurog2 versus Th + Neurog2 + Gbx2, ns *P* = 0.9023, *t* = 0.3642, df = 39. For Ctx, *F* = 5.79, **P = 0.0062; Ctx basal (*n* = 20) versus Ctx + Neurog2 (*n* = 15), **P = 0.0076, *t* = 3.222, df = 40; Ctx basal versus Ctx + Neurog2 + Gbx2 (*n* = 8), ns *P* = 0.9186, *t* = 0.1029, df = 40; Ctx + Neurog2 versus Ctx + Neurog2 + Gbx2, *P = 0.0403, *t* = 2.416, df = 40. *Ctip2*, for Th, *F* = 15.57, ***P < 0.0001; Th basal (*n* = 19) versus Th + Neurog2 (*n* = 14), ***P < 0.0001, *t* = 5.452, df = 37; Th basal versus Th + Neurog2 + Gbx2 (*n* = 7), ns *P* = 0.4738, *t* = 0.7238, df = 37; Th + Neurog2 versus Th + Neurog2 + Gbx2, **P = 0.0028, *t* = 3.457, df = 37. For Ctx, *F* = 4.681, *P = 0.0154; Ctx basal (*n* = 20) versus Ctx + Neurog2 (*n* = 12), *P = 0.018, *t* = 2.913, df = 37; Ctx basal versus Ctx + Neurog2 + Gbx2 (*n* = 8), ns *P* = 0.9460, *t* = 0.06815, df = 37; Ctx + Neurog2 versus Ctx + Neurog2 + Gbx2, *P = 0.05, *t* = 2.268, df = 37. *Ror*α, for Th, *F* = 0.7022, ns *P* = 0.5015; Th basal (*n* = 22) versus Th + Neurog2 (*n* = 14), ns *P* = 0.5676, *t* = 1.183, df = 40; Th basal versus Th + Neurog2 + Gbx2 (*n* = 7), ns *P* = 0.8707, *t* = 0.4299, df = 40; Th + Neurog2 versus Th + Neurog2 + Gbx2, ns *P* = 0.8707, *t* = 0.4707, df = 40. For Ctx, *F* = 0.05697, ns *P* = 0.9447; Ctx basal (*n* = 22) versus Ctx + Neurog2 (*n* = 14), ns *P* = 0.9827, *t* = 0.1035, df = 41; Ctx basal versus Ctx + Neurog2 + Gbx2 (*n* = 8), ns *P* = 0.9827, *t* = 0.2709, df = 41; Ctx + Neurog2 versus Ctx + Neurog2 + Gbx2, ns *P* = 0.9827, *t* = 0.3321, df = 41. *Lef1*, for Th, *F* = 0.2178, ns *P* = 0.8053; Th basal (*n* = 21) versus Th + Neurog2 (*n* = 14), ns *P* = 0.8893, *t* = 0.6494, df = 40; Th basal versus Th + Neurog2 + Gbx2 (*n* = 8), ns *P* = 0.9353, *t* = 0.3267, df = 40; Th + Neurog2 versus Th + Neurog2 + Gbx2, ns *P* = 0.9353, *t* = 0.1993, df = 40. For Ctx, *F* = 0.4896, ns *P* = 0.6164; Ctx basal (*n* = 22) versus Ctx + Neurog2 (*n* = 14), ns *P* = 0.7079, *t* = 0.9725, df = 41; Ctx basal versus Ctx + Neurog2 + Gbx2 (*n* = 8), ns *P* = 0.8896, *t* = 0.1396, df = 41; Ctx + Neurog2 versus Ctx + Neurog2 + Gbx2, ns *P* = 0.7871, *t* = 0.6201, df = 41. *Fezf2*, for Th, *F* = 21.11, ***P < 0.0001; Th basal (*n* = 17) versus Th + Neurog2 (*n* = 10), ***P < 0.0001, *t* = 5.764, df = 32; Th basal versus Th + Neurog2 + Gbx2 (*n* = 8), ***P < 0.0001, *t* = 4.800, df = 32; Th + Neurog2 versus Th + Neurog2 + Gbx2, ns *P* = 0.6177, *t* = 0.5040, df = 32. For Ctx, *F* = 0.2050, ns *P* = 0.8157; Ctx basal (*n* = 18) versus Ctx + Neurog2 (*n* = 10), ns *P* = 0.9051, *t* = 0.3393, df = 33; Ctx basal versus Ctx + Neurog2 + Gbx2 (*n* = 8), ns *P* = 0.9051, *t* = 0.3998, df = 33; Ctx + Neurog2 versus Ctx + Neurog2 + Gbx2, ns *P* = 0.8938, *t* = 0.6402, df = 33. *Slc17a6*, for Th, *F* = 4.011, *P = 0.0261; Th basal (*n* = 21) versus Th + Neurog2 (*n* = 13), *P = 0.0228, *t* = 2.813, df = 39; Th basal versus Th + Neurog2 + Gbx2 (*n* = 8), ns *P* = 0.4043, *t* = 1.224, df = 39; Th + Neurog2 versus Th + Neurog2 + Gbx2, ns *P* = 0.4043, *t* = 1.077, df = 39. For Ctx, *F* = 3.454, *P = 0.0427; Ctx basal (*n* = 20) versus Ctx + Neurog2 (*n* = 9), ns *P* = 0.4808, *t* = 0.7127, df = 35; Ctx basal versus Ctx + Neurog2 + Gbx2 (*n* = 9), ns *P* = 0.0702, *t* = 2.184, df = 35; Ctx + Neurog2 versus Ctx + Neurog2 + Gbx2, ns *P* = 0.0550, *t* = 2.466, df = 35.

In Fig. 6D, for the log_2_ of the ratio of H3K4me3/HeK27me3, unpaired Student’s *t* test two-tailed test; *Gbx2*, Th (*n* = 14) versus Ctx (*n* = 12), ***P< 0.0001, *t* = 8.037, df = 24; *Ror*α, Th (*n* = 23) versus Ctx (*n* = 22), *P = 0.0450, *t* = 2.065, df = 43; *Lef1*, Th (*n* = 20) versus Ctx (*n* = 21), *P = 0.0126, *t* = 2.616, df = 39; *Fezf2*, Th (*n* = 10) versus Ctx (*n* = 10), ns *P* = 0.3111, *t* = 1.042, df = 18; *Slc17a6*, Th (*n* = 16) versus Ctx (*n* = 16), ns *P* = 0.2250, *t* = 1.239, df = 30; *Pou2f2*, Th (*n* = 16) versus Ctx (*n* = 18), ns *P* = 0.5076, *t* = 0.6700, df = 32; *Tbr1*, Th (*n* = 19) versus Ctx (*n* = 21), *P = 0.0152, *t* = 2.542, df = 38; *Ctip2*, Th (*n* = 17) versus Ctx (*n* = 18), **P = 0.0013, *t* = 3.524, df = 33. For the expression levels (1/ΔCt), unpaired Student’s *t* test two-tailed test; *Gbx2*, Th (*n* = 19) versus Ctx (*n* = 14), ***P < 0.0001, *t* = 9.066, df = 31; *Ror*α, Th (*n* = 22) versus Ctx (*n* = 14), *P = 0.0216, *t* = 2.409, df = 34; *Lef1*, Th (*n* = 22) versus Ctx (*n* = 22), ***P < 0.0001, *t* = 6.388, df = 42; *Fezf2*, Th (*n* = 10) versus Ctx (*n* = 10), **P = 0.0028, *t* = 3.458, df = 18; *Slc17a6*, Th (*n* = 21) versus Ctx (*n* = 14), *P = 0.0298, *t* = 2.271, df = 33; *Pou2f2*, Th (*n* = 17) versus Ctx (*n* = 11), *P = 0.0118, *t* = 2.708, df = 26; *Tbr1*, Th (*n* = 13) versus Ctx (*n* = 20), ns *P* = 0.3033, *t* = 1.047, df = 31; *Ctip2*, Th (*n* = 14) versus Ctx (*n* = 14), ns *P* = 0.1874, *t* = 3.524, df = 26.

In Fig. 6E, for the epigenetics, ordinary one-way ANOVA test with Holm-Sidak’s multiple comparisons test. For *Sp9*, *F* = 6.486, **P = 0.0036; dLG versus VPM, **P = 0.0018, *n* = 14, *t* = 3.587, df = 41; dLG versus MGv, *P = 0.0394, *n* = 14 to 16, *t* = 2.128, df = 41. *Hs6st2*, *F* = 4.188, *P = 0.0215; dLG versus VPM, *P = 0.0164, *n* = 13 to 18, *t* = 2.764, df = 45; dLG versus MGv, *P = 0.0268, *n* = 13 to 17, *t* = 2.289, df = 45. *Crabp2*, *F* = 4.794, *P = 0.0132; MGv versus dLG, *P = 0.0409, *n* = 18 to 12, *t* = 2.108, df = 43; MGv versus VPM, *P = 0.01, *n* = 16 to 18, *t* = 2.958, df = 43. *Tshz1*, *F* = 5.125, *P = 0.0106; MGv versus dLG, *P = 0.0355, *n* = 13 to 15, *t* = 2.178, df = 39; MGv versus VPM, **P = 0.0072, *n* = 14 to 15, *t* = 3.098, df = 39. *Cck*, *F* = 5.489, **P = 0.0076; VPM versus dLG, *P = 0.0227, *n* = 13 to 16, *t* = 2.365, df = 42; VPM versus MGv, **P = 0.0058, *n* = 16, *t* = 3.164, df = 42. For in vivo basal expression, data from the RNA-seq analysis of the astrocytes (adjusted *P* < 0.1, log_2_FC > 0.322) from the three thalamic nuclei were used.

For fig. S1H, Pearson correlation coefficient, *R* = 0.9750593 (*T* = 121.83, df = 769, ***P < 2.2 × 10^−16^). For fig. S3C, for the differential expression of astrocytes, Wilcoxon rank sum test (adj. *P* < 0.1, log_2_FC > 0.1, As-Th: 549 DEGs, As-Ctx: 1106 DEGs). For the differential expression of neurons, Wilcoxon rank sum test (adj. *P* < 0.1, log_2_FC > 0.1, Ns-Th: 2425 DEGs, Ns-Ctx: 1845 DEGs) (table S2). For fig. S3D, hypergeometric test (one-sided Fisher’s exact test). As-Th enriched in Ns-Th, ***P = 4.001415 × 10^−73^, OD = 5.57147; As-Th enriched in Ns-Ctx, ns *P =* 0.9992975, OD = 0.6456831; As-Ctx enriched in Ns-Ctx, ***P = 2.066775 × 10^−159^, OD = 7.142444; As-Ctx enriched in Ns-Th, ns *P =* 0.9962708, OD = 0.7517044. Quantification was recovered from data of scRNA-seq analysis of astrocytic DEGs (adj. *P* < 0.1, log_2_FC > 0.1, As-Th: 549 DEGs, As-Ctx: 1106 DEGs), neuronal DEGs (adj. *P* < 0.1, log_2_FC > 0.1, Ns-Th: 2425 DEGs, Ns-Ctx: 1845 DEGs), and among both cell types in thalamic DEGs (adj. *P* < 0.1, log_2_FC > 0.1, Ns-Th: 3991 DEGs, As-Th: 1642 DEGs) and cortical DEGs (adj. *P* < 0.1, log_2_FC > 0.1, Ns-Ctx: 4142 DEGs, As-Th: 1562 DEGs) (table S2). For fig. S3 (F and G), significance values according to DE analysis performed in Fig. 2E and fig. S3C (table S2). For fig. S4E, Student’s unpaired two-tailed *t* test; *Slc17a6*, **P = 0.0018, *n* = 14; *Ror*α, *P = 0.0124, *n* = 14; *Lef1*, ***P < 0.0001, *n* = 11 to 14; *Gbx2*, ***P < 0.0001, *n* = 11 to 14; *Pou2f2*, *P = 0.0382, *n* = 12 to 14; *Tcf7l2*, ***P = 0.0003, *n* = 8; *Zic1*, *P = 0.0342, *n* = 8; *Foxg1*, ***P < 0.0001, *n* = 8; *Meis2*, ***P < 0.0001, *n* = 8; *Fezf2*, ***P = 0.0007, *n* = 9.

For fig. S5D, for the differential expression of astrocytes, Wilcoxon rank sum test (adj. *P* < 0.1, log_2_FC > 0.1; 426-DEG As-Ctx1, 258-DEG As-Ctx2, 325-DEG As-Ctx3) and neuronal DEGs (adj. *P* < 0.1, log_2_FC > 0.1; 567-DEG Ns-Ctx1, 335-DEG Ns-Ctx2, 980-DEG Ns-Ctx3) between distinct cortical regions (table S5). For fig. S5E, hypergeometric test (one-sided Fisher’s exact test) from intersect between the populations of genes for the comparison of significant overexpression in As-Ctx1 and enriched in Ns-Ctx1, ***P = 7.82738 × 10^−213^, OD = 47.36863; significant overexpression in As-Ctx2 and enriched in Ns-Ctx1, ***P = 1.633766 × 10^−33^, OD = 9.798625; significant overexpression in As-Ctx3 and enriched in Ns-Ctx1, ns *P* = 0.1058762, OD = 1.462861; significant overexpression in As-Ctx1 and enriched in Ns-Ctx2, ***P = 7.232017 × 10^−63^, OD = 16.83166; significant overexpression in As-Ctx2 and enriched in Ns-Ctx2, ***P = 1.007917 × 10^−95^, OD = 39.70291; significant overexpression in As-Ctx3 and enriched in Ns-Ctx2, ns *P* = 70.9493607, OD = 0.4745243; significant overexpression in As-Ctx1 and enriched in Ns-Ctx3, ns *P* = 0.9978754, OD = 0.4842368; significant overexpression in As-Ctx2 and enriched in Ns-Ctx3, ns *P* = 0.9966557, OD = 0.3993004; significant overexpression in As-Ctx3 and enriched in Ns-Ctx3, ***P = 7.169234 × 10^−93^, OD = 15.39014.

For fig. S6B, left graph, ordinary one-way ANOVA and Holm-Sidak’s multiple comparisons test, *F* = 0.07668, ns *P* = 0.9266; dLG versus VPM, ns *P* = 0.9737; dLG versus MGv, ns *P* = 0.9737; MGv versus VPM, ns *P* = 0.9737, *n* = 5. Right graph, ordinary one-way ANOVA and Holm-Sidak’s multiple comparisons test, *F* = 0.3985, ns *P* = 0.6799; dLG versus VPM, ns *P* = 0.8286; dLG versus MGv, ns *P* = 0.7913; MGv versus VPM, ns *P* = 0.7913, *n* = 5 electroporated animals. In fig. S6C, left graph, *n* = 59 dLG clones, *n* = 179 VPM clones, and *n* = 82 MGv clones. In the right graph, Kruskal-Wallis test with Dunn’s multiple comparisons test. For dLG clones, *P* > 0.9999 in dLG, *P* > 0.9999 in VPM, and *P* = 0.6773 in MGv, *n* = 43 clones with 3 to 10 cells and *n* = 16 clones with >10 cells. For VPM clones, *P* = 0.6386 in dLG, *P* > 0.9999 in VPM, and *P* = 0.0976 in MGv, *n* = 137 clones with 3 to 10 cells and *n* = 44 clones with >10 cells. For MGv clones, *P* = 0.4436 in dLG, *P* > 0.9999 in VPM, and *P* > 0.9999 in MGv, *n* = 66 clones with 3 to 10 cells and *n* = 15 clones with >10 cells. In fig. S6F, Mann-Whitney *U* test nonparametric two-tailed test (*n* = 128 clones); neurons versus nonneurons, ns *P* = 0.3112. In fig. S6G, Kruskal-Wallis test, with Dunn’s multiple comparisons test. *n* = 4 electroporated animals. For dLG neuronal clones, *n* = 61 clones, ***P < 0.0001; dLG (CI: 80.53 to 88.87%) versus VPM (CI: 5.962 to 13.56%), ***P < 0.0001; dLG versus MGv (CI: 3.069 to 8.002%), ***P < 0.0001. For dLG nonneuronal clones, *n* = 14 clones, ***P < 0.0001; dLG (CI: 100 to 100%) versus VPM (CI: 0 to 0%), ***P < 0.0001; dLG versus MGv (CI: 0 to 0%), ***P < 0.0001. For VPM neuronal clones, *n* = 7 clones, ***P = 0.0007; dLG (CI: 0.856 to 37.24%) versus VPM (CI: 45.53 to 82.08%), **P = 0.0081; dLG versus MGv (CI: −0.13 to 34.42%), ***P = 0.0039. For VPM nonneuronal clones, *n* = 25 clones, ***P < 0.0001; dLG (CI: −0.7909 to 7.458%) versus VPM (CI: 92.54 to 100.8%), ***P < 0.0081; dLG versus MGv (CI: 0 to 0%), ***P < 0.0001. For MGv neuronal clones, *n* = 4 clones, **P = 0.0052; VPM (CI: −18.95 to 60.61%) versus MGv (CI: 39.39 to 118.9%), ns *P* = 0.0917; dLG (CI: 0 to 0%) versus MGv, **P = 0.0077. For MGv nonneuronal clones, *n* = 3 clones, *P = 0.0357; VPM (CI: 0 to 0%) versus MGv (CI: 100 to 100%), *P = 0.0286; dLG (CI: 0 to 0%) versus MGv, **P = 0.0286. For fig. S7B, in Th, **P = 0.0079, *n* = 5 injected mice (427 cells with control and 572 cells with *Neurog2* virus), and in Cx, **P = 0.0043, *n* = 5 injected mice (362 cells with control and 292 cells with *Neurog2* virus).

For fig. S8F, for *Gfap*, two-tailed unpaired Student’s *t* test, **P = 0.0014, *n* = 8; for *Neurog2,* ordinary one-way ANOVA test, *F* = 23.41, ***P < 0.0001; Holm-Sidak’s multiple comparisons test, Tuj1^+^/RFP^+^ versus RFP^+^, ***P = 0.0006; Tuj1^+^/RFP^+^ versus Tuj1^−^/RFP^−^, ***P < 0.0001; RFP^+^ versus Tuj1^−^/RFP^−^, *P = 0.0116; *n* = 12 cultures. For fig. S8G, Student’s *t* test or Mann-Whitney *U* test nonparametric two-tailed test; *Slc17a6*, **P = 0.0058, *n* = 16; *Ror*α, **P = 0.0027, *n* = 17; *Gbx2*, **P = 0.0065, *n* = 7; *Pou2f2*, *P = 0.0181, *n* = 5; *Lef1*, **P = 0.0016, *n* = 7; *Tbr1*, **P = 0.0078, *n* = 11; *Ctip2*, **P = 0.0079, *n* = 5.

For fig. S10B, for *Gapdh* and *Cdx2* upper graphs, two-tailed unpaired Student’s *t* test; for *Gapdh*, **P = 0.0065, *n* = 9; and for *Cdx2*, ns *P* = 0.3406, *n* = 9. In the lower graph, *Gapdh* versus *Cdx2*, Mann-Whitney *U* test nonparametric two-tailed test, ***P < 0.0001, *n* = 8. For *Gbx2*, two-tailed paired Student’s *t* test: Th, ***P < 0.0001, *n* = 14; Ctx, **P = 0.0049, *n* = 12. For *Ror*α, two-tailed Wilcoxon matched pairs test: Th, ***P < 0.0001, *n* = 23; Ctx, *P = 0.019, *n* = 22. For *Lef1*, two-tailed paired Student’s *t* test: Th, ns *P* = 0.0563, *n* = 18; Ctx, ns *P* = 0.211, *n* = 23. For *Fezf2*, two-tailed paired Student’s *t* test: Th, ns *P* = 0.2506, *n* = 9; Ctx, ns *P* = 0.3506, *n* = 10. For *Slc17a6*, two-tailed Wilcoxon matched pairs test: Th, *P = 0.0214, *n* = 16; Ctx, ***P = 0.0003, *n* = 16. For *Pou2f2*, two-tailed Wilcoxon matched pairs test: Th, ns *P* = 0.7819, *n* = 17; Ctx, ns *P* = 0.2121, *n* = 18. For *Tbr1*, two-tailed paired Student’s *t* test: Th, ***P = 0.0003, *n* = 19; Ctx, ns *P* = 0.1748, *n* = 21. For *Ctip2*, two-tailed paired Student’s *t* test: Th, *P = 0.0156, *n* = 17; Ctx, ns *P* = 0.1471, *n* = 18. For fig. S10D, *Sp9*, two-tailed paired Student’s *t* test: dLG, **P = 0.0045, *n* = 14; VPM, ***P = 0.0001, *n* = 14; and MGv, ***P < 0.0001, *n* = 16. *Hs6st2*, two-tailed paired Student’s *t* test: dLG, **P = 0.0031, *n* = 14; VPM, ***P = 0.0001, *n* = 18; and MGv, ***P < 0.0001, *n* = 18. *Cck*, two-tailed paired Student’s *t* test: dLG, ***P < 0.0001, *n* = 15; VPM, ***P < 0.0001, *n* = 18; and MGv, ***P < 0.0001, *n* = 18. *Crabp2*, two-tailed Wilcoxon matched pairs test: dLG, *P = 0.0245, *n* = 14; VPM, ns *P* = 0.0987, *n* = 18; and MGv, ***P < 0.0001, *n* = 19. *Tshz1*, two-tailed Wilcoxon matched pairs test: dLG, ***P < 0.0001, *n* = 13; VPM, ***P = 0.0001, *n* = 14; and MGv, ***P < 0.0001, *n* = 16.

## SUPPLEMENTARY MATERIALS

Supplementary material for this article is available at http://advances.sciencemag.org/cgi/content/full/7/15/eabe8978/DC1

## Appendix

View/request a protocol for this paper from *Bio-protocol*.

## References

[1] V. Metzis, S. Steinhauser, E. Pakanavicius, M. Gouti, D. Stamataki, K. Ivanovitch, T. Watson, T. Rayon, S. N. M. Gharavy, R. Lovell-Badge, N. M. Luscombe, J. Briscoe, Nervous system regionalization entails axial allocation before neural differentiation. Cell 175, 1105–1118.e17 (2018). 30343898 10.1016/j.cell.2018.09.040 PMC6218657

[2] D. Mi, Z. Li, L. Lim, M. Li, M. Moissidis, Y. Yang, T. Gao, T. X. Hu, T. Pratt, D. J. Price, N. Sestan, O. Marín, Early emergence of cortical interneuron diversity in the mouse embryo. Science 360, 81–85 (2018). 29472441 10.1126/science.aar6821 PMC6195193

[3] C. Mayer, C. Hafemeister, R. C. Bandler, R. Machold, R. Batista Brito, X. Jaglin, K. Allaway, A. Butler, G. Fishell, R. Satija, Developmental diversification of cortical inhibitory interneurons. Nature 555, 457–462 (2018). 29513653 10.1038/nature25999 PMC6052457

[4] L. Telley, G. Agirman, J. Prados, N. Amberg, S. Fièvre, P. Oberst, G. Bartolini, I. Vitali, C. Cadilhac, S. Hippenmeyer, L. Nguyen, A. Dayer, D. Jabaudon, Temporal patterning of apical progenitors and their daughter neurons in the developing neocortex. Science 364, eaav2522 (2019). 31073041 10.1126/science.aav2522

[5] S. Lodato, P. Arlotta, Generating neuronal diversity in the mammalian cerebral cortex. Annu. Rev. Cell Dev. Biol. 31, 699–720 (2015). 26359774 10.1146/annurev-cellbio-100814-125353 PMC4778709

[6] T. M. Jessell, Neuronal specification in the spinal cord: Inductive signals and transcriptional codes. Nat. Rev. Genet. 1, 20–29 (2000). 11262869 10.1038/35049541

[7] L. C. Greig, M. B. Woodworth, M. J. Galazo, H. Padmanabhan, J. D. Macklis, Molecular logic of neocortical projection neuron specification, development and diversity. Nat. Rev. Neurosci. 14, 755–769 (2013). 24105342 10.1038/nrn3586 PMC3876965

[8] T. J. Nowakowski, A. Bhaduri, A. A. Pollen, B. Alvarado, M. A. Mostajo-Radji, E. di Lullo, M. Haeussler, C. Sandoval-Espinosa, S. J. Liu, D. Velmeshev, J. R. Ounadjela, J. Shuga, X. Wang, D. A. Lim, J. A. West, A. A. Leyrat, W. J. Kent, A. R. Kriegstein, Spatiotemporal gene expression trajectories reveal developmental hierarchies of the human cortex. Science 358, 1318–1323 (2017). 29217575 10.1126/science.aap8809 PMC5991609

[9] D. H. Rowitch, A. R. Kriegstein, Developmental genetics of vertebrate glial-cell specification. Nature 468, 214–222 (2010). 21068830 10.1038/nature09611

[10] C. Hochstim, B. Deneen, A. Lukaszewicz, Q. Zhou, D. J. Anderson, Identification of positionally distinct astrocyte subtypes whose identities are specified by a homeodomain code. Cell 133, 510–522 (2008). 18455991 10.1016/j.cell.2008.02.046 PMC2394859

[11] A. V. Molofsky, K. W. Kelley, H. H. Tsai, S. A. Redmond, S. M. Chang, L. Madireddy, J. R. Chan, S. E. Baranzini, E. M. Ullian, D. H. Rowitch, Astrocyte-encoded positional cues maintain sensorimotor circuit integrity. Nature 509, 189–194 (2014). 24776795 10.1038/nature13161 PMC4057936

[12] J. García-Marqués, L. López-Mascaraque, Clonal identity determines astrocyte cortical heterogeneity. Cereb. Cortex 23, 1463–1472 (2013). 22617854 10.1093/cercor/bhs134

[13] O. A. Bayraktar, T. Bartels, S. Holmqvist, V. Kleshchevnikov, A. Martirosyan, D. Polioudakis, L. B. Haim, A. M. H. Young, M. Y. Batiuk, K. Prakash, A. Brown, K. Roberts, M. F. Paredes, R. Kawaguchi, J. H. Stockley, K. Sabeur, S. M. Chang, E. Huang, P. Hutchinson, E. M. Ullian, M. Hemberg, G. Coppola, M. G. Holt, D. H. Geschwind, D. H. Rowitch, Astrocyte layers in the mammalian cerebral cortex revealed by a single-cell in situ transcriptomic map. Nat. Neurosci. 23, 500–509 (2020). 32203496 10.1038/s41593-020-0602-1 PMC7116562

[14] M. Y. Batiuk, A. Martirosyan, J. Wahis, F. de Vin, C. Marneffe, C. Kusserow, J. Koeppen, J. F. Viana, J. F. Oliveira, T. Voet, C. P. Ponting, T. G. Belgard, M. G. Holt, Identification of region-specific astrocyte subtypes at single cell resolution. Nat. Commun. 11, 1220–1215 (2020). 32139688 10.1038/s41467-019-14198-8 PMC7058027

[15] B. S. Khakh, B. Deneen, The emerging nature of astrocyte diversity. Annu. Rev. Neurosci. 42, 187–207 (2019). 31283899 10.1146/annurev-neuro-070918-050443

[16] C. Nolte, M. Matyash, T. Pivneva, C. G. Schipke, C. Ohlemeyer, U. K. Hanisch, F. Kirchhoff, H. Kettenmann, GFAP promoter-controlled EGFP-expressing transgenic mice: A tool to visualize astrocytes and astrogliosis in living brain tissue. Glia 33, 72–86 (2001). 11169793

[17] W.-P. Ge, A. Miyawaki, F. H. Gage, Y. N. Jan, L. Y. Jan, Local generation of glia is a major astrocyte source in postnatal cortex. Nature 484, 376–380 (2012). 22456708 10.1038/nature10959 PMC3777276

[18] L. Chen, Q. Guo, J. Y. H. Li, Transcription factor Gbx2 acts cell-nonautonomously to regulate the formation of lineage-restriction boundaries of the thalamus. Development 136, 1317–1326 (2009). 19279136 10.1242/dev.030510 PMC2687463

[19] B. Tasic, Z. Yao, L. T. Graybuck, K. A. Smith, T. N. Nguyen, D. Bertagnolli, J. Goldy, E. Garren, M. N. Economo, S. Viswanathan, O. Penn, T. Bakken, V. Menon, J. Miller, O. Fong, K. E. Hirokawa, K. Lathia, C. Rimorin, M. Tieu, R. Larsen, T. Casper, E. Barkan, M. Kroll, S. Parry, N. V. Shapovalova, D. Hirschstein, J. Pendergraft, H. A. Sullivan, T. K. Kim, A. Szafer, N. Dee, P. Groblewski, I. Wickersham, A. Cetin, J. A. Harris, B. P. Levi, S. M. Sunkin, L. Madisen, T. L. Daigle, L. Looger, A. Bernard, J. Phillips, E. Lein, M. Hawrylycz, K. Svoboda, A. R. Jones, C. Koch, H. Zeng, Shared and distinct transcriptomic cell types across neocortical areas. Nature 563, 72–78 (2018). 30382198 10.1038/s41586-018-0654-5 PMC6456269

[20] B. J. Molyneaux, L. A. Goff, A. C. Brettler, H.-H. Chen, J. R. Brown, S. Hrvatin, J. L. Rinn, P. Arlotta, DeCoN: Genome-wide analysis of in vivo transcriptional dynamics during pyramidal neuron fate selection in neocortex. Neuron 85, 275–288 (2015). 25556833 10.1016/j.neuron.2014.12.024 PMC4430475

[21] J. D. Cahoy, B. Emery, A. Kaushal, L. C. Foo, J. L. Zamanian, K. S. Christopherson, Y. Xing, J. L. Lubischer, P. A. Krieg, S. A. Krupenko, W. J. Thompson, B. A. Barres, A transcriptome database for astrocytes, neurons, and oligodendrocytes: A new resource for understanding brain development and function. J. Neurosci. 28, 264–278 (2008). 18171944 10.1523/JNEUROSCI.4178-07.2008 PMC6671143

[22] H. Gezelius, V. Moreno-Juan, C. Mezzera, S. Thakurela, L. M. Rodríguez-Malmierca, J. Pistolic, V. Benes, V. K. Tiwari, G. López-Bendito, Genetic labeling of nuclei-specific thalamocortical neurons reveals putative sensory-modality specific genes. Cereb. Cortex 27, 5054–5069 (2017). 27655933 10.1093/cercor/bhw290 PMC7610997

[23] A. Zeisel, H. Hochgerner, P. Lönnerberg, A. Johnsson, F. Memic, J. van der Zwan, M. Häring, E. Braun, L. E. Borm, G. L. Manno, S. Codeluppi, A. Furlan, K. Lee, N. Skene, K. D. Harris, J. Hjerling-Leffler, E. Arenas, P. Ernfors, U. Marklund, S. Linnarsson, Molecular architecture of the mouse nervous system. Cell 174, 999–1014.e22 (2018). 30096314 10.1016/j.cell.2018.06.021 PMC6086934

[24] A. Baser, M. Skabkin, S. Kleber, Y. Dang, G. S. Gülcüler Balta, G. Kalamakis, M. Göpferich, D. C. Ibañez, R. Schefzik, A. S. Lopez, E. L. Bobadilla, C. Schultz, B. Fischer, A. Martin-Villalba, Onset of differentiation is post-transcriptionally controlled in adult neural stem cells. Nature 566, 100–104 (2019). 30700908 10.1038/s41586-019-0888-x

[25] M. Figueres-Oñate, J. García-Marqués, L. López-Mascaraque, UbC-*StarTrack*, a clonal method to target the entire progeny of individual progenitors. Sci. Rep. 6, 33896 (2016). 27654510 10.1038/srep33896 PMC5031994

[26] W. Shi, A. Xianyu, Z. Han, X. Tang, Z. Li, H. Zhong, T. Mao, K. Huang, S. H. Shi, Ontogenetic establishment of order-specific nuclear organization in the mammalian thalamus. Nat. Neurosci. 20, 516–528 (2017). 28250409 10.1038/nn.4519 PMC5374008

[27] S. Z. H. Wong, E. P. Scott, W. Mu, X. Guo, E. Borgenheimer, M. Freeman, G. L. Ming, Q. F. Wu, H. Song, Y. Nakagawa, In vivo clonal analysis reveals spatiotemporal regulation of thalamic nucleogenesis. PLOS Biol. 16, e2005211 (2018). 29684005 10.1371/journal.pbio.2005211 PMC5933804

[28] S. Gascón, E. Murenu, G. Masserdotti, F. Ortega, G. L. Russo, D. Petrik, A. Deshpande, C. Heinrich, M. Karow, S. P. Robertson, T. Schroeder, J. Beckers, M. Irmler, C. Berndt, J. P. F. Angeli, M. Conrad, B. Berninger, M. Götz, Identification and successful negotiation of a metabolic checkpoint in direct neuronal reprogramming. Cell Stem Cell 18, 396–409 (2016). 26748418 10.1016/j.stem.2015.12.003

[29] C. Mallika, Q. Guo, J. Y. H. Li, *Gbx2* is essential for maintaining thalamic neuron identity and repressing habenular characters in the developing thalamus. Dev. Biol. 407, 26–39 (2015). 26297811 10.1016/j.ydbio.2015.08.010 PMC4641819

[30] N. Mattugini, R. Bocchi, V. Scheuss, G. L. Russo, O. Torper, C. L. Lao, M. Götz, Inducing different neuronal subtypes from astrocytes in the injured mouse cerebral cortex. Neuron 103, 1086–1095.e5 (2019). 31488328 10.1016/j.neuron.2019.08.009 PMC6859713

[31] B. Mahato, K. D. Kaya, Y. Fan, N. Sumien, R. A. Shetty, W. Zhang, D. Davis, T. Mock, S. Batabyal, A. Ni, S. Mohanty, Z. Han, R. Farjo, M. J. Forster, A. Swaroop, S. H. Chavala, Pharmacologic fibroblast reprogramming into photoreceptors restores vision. Nature 581, 83–88 (2020). 32376950 10.1038/s41586-020-2201-4 PMC7469946

[32] K. K. Bluske, T. Y. Vue, Y. Kawakami, M. M. Taketo, K. Yoshikawa, J. E. Johnson, Y. Nakagawa, β-Catenin signaling specifies progenitor cell identity in parallel with Shh signaling in the developing mammalian thalamus. Development 139, 2692–2702 (2012). 22745311 10.1242/dev.072314 PMC3392701

[33] S. Lodato, B. J. Molyneaux, E. Zuccaro, L. A. Goff, H.-H. Chen, W. Yuan, A. Meleski, E. Takahashi, S. Mahony, J. L. Rinn, D. K. Gifford, P. Arlotta, Gene co-regulation by *Fezf2* selects neurotransmitter identity and connectivity of corticospinal neurons. Nat. Neurosci. 17, 1046–1054 (2014). 24997765 10.1038/nn.3757 PMC4188416

[34] A. B. Mihalas, R. F. Hevner, Control of neuronal development by T-box genes in the brain. Curr. Top. Dev. Biol. 122, 279–312 (2017). 28057268 10.1016/bs.ctdb.2016.08.001

[35] A. Y.-S. Huang, J. Woo, D. Sardar, B. Lozzi, N. A. Bosquez Huerta, C.-C. J. Lin, D. Felice, A. Jain, A. Paulucci-Holthauzen, B. Deneen, Region-specific transcriptional control of astrocyte function oversees local circuit activities. Neuron 106, 992–1008.e9 (2020). 32320644 10.1016/j.neuron.2020.03.025 PMC7879989

[36] Q. Guo, J. Y. H. Li, Defining developmental diversification of diencephalon neurons through single cell gene expression profiling. Development 146, dev174284 (2019). 30872278 10.1242/dev.174284 PMC6602344

[37] Y. Hirabayashi, N. Suzki, M. Tsuboi, T. A. Endo, T. Toyoda, J. Shinga, H. Koseki, M. Vidal, Y. Gotoh, Polycomb limits the neurogenic competence of neural precursor cells to promote astrogenic fate transition. Neuron 63, 600–613 (2009). 19755104 10.1016/j.neuron.2009.08.021

[38] Y. Hirabayashi, Y. Gotoh, Epigenetic control of neural precursor cell fate during development. Nat. Rev. Neurosci. 11, 377–388 (2010). 20485363 10.1038/nrn2810

[39] K. Kim, A. Doi, B. Wen, K. Ng, R. Zhao, P. Cahan, J. Kim, M. J. Aryee, H. Ji, L. I. R. Ehrlich, A. Yabuuchi, A. Takeuchi, K. C. Cunniff, H. Hongguang, S. Mckinney-Freeman, O. Naveiras, T. J. Yoon, R. A. Irizarry, N. Jung, J. Seita, J. Hanna, P. Murakami, R. Jaenisch, R. Weissleder, S. H. Orkin, I. L. Weissman, A. P. Feinberg, G. Q. Daley, Epigenetic memory in induced pluripotent stem cells. Nature 467, 285–290 (2010). 20644535 10.1038/nature09342 PMC3150836

[40] J. P. Magnusson, C. Goritz, J. Tatarishvili, D. O. Dias, E. M. K. Smith, O. Lindvall, Z. Kokaia, J. Frisen, A latent neurogenic program in astrocytes regulated by Notch signaling in the mouse. Science 346, 237–241 (2014). 25301628 10.1126/science.346.6206.237

[41] G. Nato, A. Caramello, S. Trova, V. Avataneo, C. Rolando, V. Taylor, A. Buffo, P. Peretto, F. Luzzati, Striatal astrocytes produce neuroblasts in an excitotoxic model of Huntington’s disease. Development 142, 840–845 (2015). 25655705 10.1242/dev.116657

[42] H. Qian, X. Kang, J. Hu, D. Zhang, Z. Liang, F. Meng, X. Zhang, Y. Xue, R. Maimon, S. F. Dowdy, N. K. Devaraj, Z. Zhou, W. C. Mobley, D. W. Cleveland, X. D. Fu, Reversing a model of Parkinson’s disease with in situ converted nigral neurons. Nature 582, 550–556 (2020). 32581380 10.1038/s41586-020-2388-4 PMC7521455

[43] N. Tamamaki, Y. Yanagawa, R. Tomioka, J.-I. Miyazaki, K. Obata, T. Kaneko, Green fluorescent protein expression and colocalization with calretinin, parvalbumin, and somatostatin in the GAD67-GFP knock-in mouse. J. Comp. Neurol. 467, 60–79 (2003). 14574680 10.1002/cne.10905

[44] N. Antón-Bolaños, A. Sempere-Ferràndez, T. Guillamón-Vivancos, F. J. Martini, L. Pérez-Saiz, H. Gezelius, A. Filipchuk, M. Valdeolmillos, G. López-Bendito, Prenatal activity from thalamic neurons governs the emergence of functional cortical maps in mice. Science 364, 987–990 (2019). 31048552 10.1126/science.aav7617 PMC7611000

[45] M. Scandaglia, J. P. Lopez-Atalaya, A. Medrano-Fernandez, M. T. Lopez-Cascales, B. del Blanco, M. Lipinski, E. Benito, R. Olivares, S. Iwase, Y. Shi, A. Barco, Loss of Kdm5c causes spurious transcription and prevents the fine-tuning of activity-regulated enhancers in neurons. Cell Rep. 21, 47–59 (2017). 28978483 10.1016/j.celrep.2017.09.014 PMC5679733

[46] Y. Liao, G. K. Smyth, W. Shi, featureCounts: An efficient general purpose program for assigning sequence reads to genomic features. Bioinformatics 30, 923–930 (2014). 24227677 10.1093/bioinformatics/btt656

[47] A. Zhu, J. G. Ibrahim, M. I. Love, Heavy-tailed prior distributions for sequence count data: Removing the noise and preserving large differences. Bioinformatics 35, 2084–2092 (2019). 30395178 10.1093/bioinformatics/bty895 PMC6581436

[48] G. Yu, L.-G. Wang, Y. Han, Q.-Y. He, clusterProfiler: An R package for comparing biological themes among gene clusters. OMICS 16, 284–287 (2012). 22455463 10.1089/omi.2011.0118 PMC3339379

[49] T. Stuart, A. Butler, P. Hoffman, C. Hafemeister, E. Papalexi, W. M. Mauck III, Y. Hao, M. Stoeckius, P. Smibert, R. Satija, Comprehensive integration of single-cell data. Cell 177, 1888–1902.e21 (2019). 31178118 10.1016/j.cell.2019.05.031 PMC6687398

[50] A. Butler, P. Hoffman, P. Smibert, E. Papalexi, R. Satija, Integrating single-cell transcriptomic data across different conditions, technologies, and species. Nat. Biotechnol. 36, 411–420 (2018). 29608179 10.1038/nbt.4096 PMC6700744

[51] V. Moreno-Juan, A. Filipchuk, N. Antón-Bolaños, C. Mezzera, H. Gezelius, B. Andrés, L. Rodríguez-Malmierca, R. Susín, O. Schaad, T. Iwasato, R. Schüle, M. Rutlin, S. Nelson, S. Ducret, M. Valdeolmillos, F. M. Rijli, G. López-Bendito, Prenatal thalamic waves regulate cortical area size prior to sensory processing. Nat. Commun. 8, 14172 (2017). 28155854 10.1038/ncomms14172 PMC5296753

[52] C. Heinrich, S. Gascón, G. Masserdotti, A. Lepier, R. Sanchez, T. Simon-Ebert, T. Schroeder, M. Götz, B. Berninger, Generation of subtype-specific neurons from postnatal astroglia of the mouse cerebral cortex. Nat. Protoc. 6, 214–228 (2011). 21293461 10.1038/nprot.2010.188

[53] T. L. Bailey, M. Boden, F. A. Buske, M. Frith, C. E. Grant, L. Clementi, J. Ren, W. W. Li, W. S. Noble, MEME SUITE: Tools for motif discovery and searching. Nucleic Acids Res. 37, W202–W208 (2009). 19458158 10.1093/nar/gkp335 PMC2703892

[54] G. Yu, L.-G. Wang, Q.-Y. He, ChIPseeker: An R/Bioconductor package for ChIP peak annotation, comparison and visualization. Bioinformatics 31, 2382–2383 (2015). 25765347 10.1093/bioinformatics/btv145

[55] S. Gillotin, F. Guillemot, Micro-chromatin immunoprecipation (μChIP) protocol for real-time PCR analysis of a limited amount of cells. Bio Protoc. 6, e1846 (2016). 10.21769/BioProtoc.1846 PMC5654496 29075654

